# Spatiotemporal transcriptomics elucidates the pathogenesis of fulminant viral myocarditis

**DOI:** 10.1038/s41392-025-02143-9

**Published:** 2025-02-10

**Authors:** Huihui Li, Xueting Chen, James Jiqi Wang, Juan Shen, Kudusi Abuduwufuer, Zhao Zhang, Zhensheng Dong, Zheng Wen, Jingwei He, Silian Chen, Wanshun Li, Chen Chen, Fan Li, Xiaodong Fang, Dao Wen Wang

**Affiliations:** 1 Division of Cardiology, Department of Internal Medicine, Tongji Hospital, Tongji Medical College, Huazhong University of Science and Technology; Hubei Key Laboratory of Genetics and Molecular Mechanisms of Cardiological Disorders, Wuhan, China; 2 BGI Research, Shenzhen, China; 3 College of Life Sciences, University of Chinese Academy of Sciences, Beijing, China; 4 Lars Bolund Institute of Regenerative Medicine Qingdao-Europe Advanced Institute for LifeSciences, BGI Research, Qingdao, China; 5 MGI Tech, Shenzhen, China; 6 BGI Research, Sanya, China; 7 State Key Laboratory of Traditional Chinese Medicine Syndrome, The Second Affiliated Hospital of Guangzhou University of Chinese Medicine, Guangzhou, China

**Keywords:** Cardiology, Infection

## Abstract

Fulminant myocarditis (FM) is a severe inflammatory condition of the myocardium that often results in sudden death, particularly in young individuals. In this study, we employed single-nucleus and spatial transcriptomics to perform a comprehensive analysis of coxsackievirus B3 (CVB3)-induced FM in A/J mice, spanning seven distinct time points pre- and post-treatment. Our findings reveal that mesothelial cells play a critical role in the early stage of myocarditis by acting as primary targets for CVB3 infection. This triggers the activation of macrophages, initiating a cascade of inflammation. Subsequently, pro-inflammatory Inflammatory_Mac and T cells infiltrate the myocardium, driving tissue damage. We also identified Cd8^+^ effector T cells as key mediators of cardiomyocyte injury. These cells release cytotoxic molecules, particularly IFN-γ, which modulates the expression of *Spi1*, a factor implicated in exacerbating cardiomyocyte death and amplifying disease progression. Therapeutic interventions targeting the IFN-γ/*Spi1* axis demonstrated significant efficacy in FM models. Notably, intravenous immunoglobulin (IVIG) treatment reduced mortality, suppressed viral proliferation, and mitigated the hyperinflammatory state of FM. IVIG therapy also downregulated IFN-γ and *Spi1* expression, underscoring its immunomodulatory and therapeutic potential. This comprehensive spatiotemporal transcriptomic analysis provides profound insights into the pathogenesis of FM and highlights actionable therapeutic targets, paving the way for more effective management strategies for this life-threatening condition.

## Introduction

Myocarditis is an inflammatory disease of the heart mostly caused by viral infections, such as coxsackievirus B3 (CVB3), parvovirus B19, adenovirus, and influenza virus.^1,2^ Other etiological factors, such as bacteria, protozoa, and fungi, as well as toxins, drugs, and systemic immune diseases, can also induce myocarditis.^3–5^ In recent years, the widespread use of antitumor drugs such as immune checkpoint inhibitors (ICIs) and the prevalence of COVID-19 have contributed to a rise in myocarditis incidence and mortality.^6–8^ Although myocarditis affects individuals of all ages, it is most common in children and young adults under 40.^9^ Fulminant myocarditis (FM) represents the most severe form of myocarditis, characterized by its abrupt onset and rapid progression to cardiac dysfunction, necessitating inotropic and mechanical circulatory support.^9^ Severe myocardial inflammation in FM can result in dilated cardiomyopathy, malignant arrhythmias, and unexpected cardiac death.^10^ Multicenter clinical studies have reported an in-hospital mortality or heart transplantation rate of 25.5% for FM patients, with a five-year survival rate below 50%.^11,12^ The mortality rate during hospitalization is even higher in pediatric cases, reaching 48%.^13^ Although life-support-based comprehensive treatment regimen has reduced the risk of in-hospital death in patients with FM to below 5%, the long-term prognosis remains poor.^14,15^

The pathogenesis of FM involves both direct viral-mediated damage and immune-mediated injury, often referred to as an “inflammatory storm”.^9^ Overactivation of the immune response following viral infection was thought to play a pivotal role in the development of FM.^16^ During the course of the disease, there is a significant inflammatory infiltration in the heart and a drastic increase in a variety of immune cells, especially pro-inflammatory macrophages and T cells. However, the detailed immune mehcanisms in FM have not been elucidated. Studying FM poses challenges due to its sudden onset, focal inflammatory lesions, low sensitivity of diagnostic tests, and the complex interplay between heterogeneous immune cells and viral interactions in cardiac tissues. The intricate network of immune and cardiac cells, coupled with the cellular, spatial, and dynamic heterogeneity of FM, complicates efforts to elucidate its molecular pathogenesis. This complexity extends even to animal models, making it difficult to determine the roles and reactions of infected versus uninfected cells in the heart. Furthermore, the understanding of innate and adaptive immune responses in FM is limited by current technological constraints. Comprehensive and unbiased characterization of cellular phenotypes, developmental trajectories, and intercellular interactions in inflamed cardiac tissues is essential to address these gaps.

Single-cell transcriptomics has emerged as a powerful tool for dissecting the complexity of cellular compositions and gene expression in diseases. Previous single-cell studies have shed light on the immune cell landscape and dynamic transcriptional activities in heart infiltrates.^17,18^ Recently, B. Han *et al*. analyzed peripheral blood mononuclear cells from children in the acute and convalescent stages of FM, demonstrating that CXCR4 blockade reduces myocardial inflammation and injury. However, the lack of spatial information and analysis at multiple time points during disease progression limits our understanding of immune mechanisms and cell-cell interactions. Spatial transcriptomics (ST), which provides a high-resolution spatial map of gene expression across tissue architecture, addresses these limitations. High-throughput spatially resolved gene expression analysis has proven invaluable in unraveling the complexity of various diseases. For instance, a prior study identified spatially restricted cell-cell interaction networks in reovirus-induced myocarditis within ileum and heart tissues.^19^ By integrating single-cell transcriptomics with ST, researchers can achieve a more comprehensive understanding of immunopathogenesis, particularly the spatial and temporal dynamics of cellular alterations during FM progression, thus identifying potential therapeutic targets.

In this study, we employed integrated single-nucleus RNA sequencing (snRNA-seq) and spatial enhanced resolution omics-sequencing (Stereo-seq) to explore the cellular and spatial heterogeneity of myocardial inflammatory processes in FM mice across multiple time intervals post-infection, both pre- and post-treatment. Our data illuminated the tropism of the virus in cardiac cells, the sequential activation of cardiac structural and immune cells, cell type-specific immune responses, and the transcriptional profiles of cell types involved in circulating immune cell recruitment and inflammatory cytokine production. These findings revealed the immune mechanisms underlying reduced cardiac function and cardiomyocyte damage in FM. Furthermore, we demonstrated the potent immunomodulatory effects of intravenous immunoglobulin (IVIG), which significantly improved cardiac function in FM patients during clinical treatment. We also uncovered new mechanisms of IVIG action, specifically its ability to suppress the IFN-γ/*Spi1* axis-mediated cardiomyocyte death. Overall, our results unveiled spatially restricted cellular interactions and host responses unique to different cell types in CVB3-induced FM. The molecular markers identified in this study provide critical insights into the underlying mechanisms of FM, paving the way for the development of novel therapeutic strategies.

## Results

### Single-nucleus and spatial transcriptome sequencing of CVB3-infected hearts

Upon systematic pathological evaluation at sequential time points post-infection, the heart functions of FM mice exhibited severe dysregulation by day 7, and the death rate was close to 60% (Supplementary Fig. 1a–c), mirroring the clinical performance of patients with FM.^20^ Histological analysis also revealed the progressive infiltration of inflammatory cells from days 1 to 7 (Supplementary Fig. 1d).

**Fig. 1:**
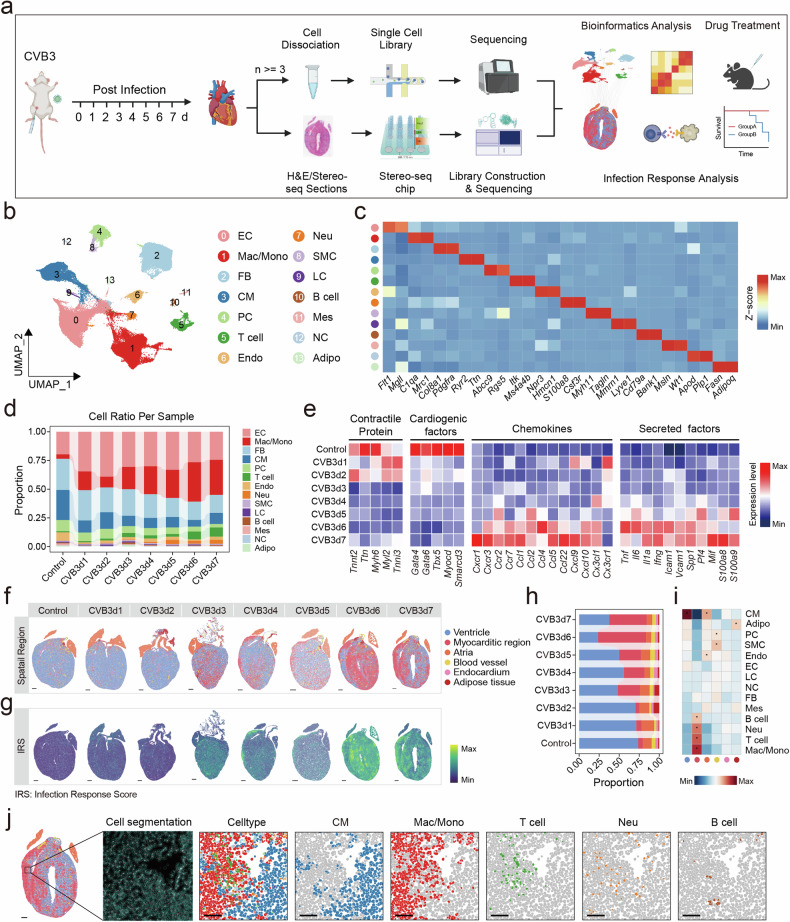
The spatiotemporal landscape of FM mice. **a** Schematic diagram of the workflow for this study. **b** UMAP embedding of all the cells colored by manually annotated cell types. **c** Heatmap showing the expression levels of cell-typing genes in each cell type. The color represents the gene expression level. **d** Cell component changes among different time points. **e** Heatmap showing the expression levels of specific functional genes at different time points. The color represents the gene expression level. The spatial structures **(f)** and infection response score (**g**) of cardiac tissue sections from control and CVB3-infected mice from 1 to 7 dpi. Scale bar: 500 μm. **h** Spatial structure proportion changes among different time points. **i** Heatmap showing the Pearson correlation between cell types defined in snRNA-seq and spatial structures defined in stereo-seq data. **j** Spatial immune cell infiltration on cardiac tissue sections from CVB3-infected mice at 7 dpi. Scale bar: 500 μm for whole cardiac section, 100 μm for zoomed view. EC endothelial cell, Mac/mono macrophage/monocyte, FB fibroblast, CM cardiomyocyte, PC pericyte, T T cell, Endo endocardial cells, Neu neutrophil, SMC smooth muscle cell, LC lymphatic cell, B B cell, Mes mesothelial cell, NC neuron cell, Adipo adipocyte

To thoroughly explore the pathophysiology of FM, we performed a comprehensive analysis for the integrated data of snRNA-seq and stereo-seq on the cardiac sections extracted from healthy and CVB3-infected A/J mice at 1–7 days post-infection (dpi) (Fig. 1a). After filtering, a total of 95,904 cells were retained for unsupervised clustering and a cell atlas encompassing 14 major cell types were identified (Fig. 1b, c, Supplementary Fig. 1e–g and Supplementary Tables 1, 2). Comprehensive cellular compositional profiling demonstrated a diminution in the proportion of cardiomyocytes and an inversely proportional surge in the immune cell fraction, with a pronounced escalation of macrophages and T lymphocytes, in temporal congruence with the advancement of the pathological state (Fig. 1d). Concurrently, the expression levels of contractile proteins and cardiogenic factors decreased, whereas those of chemokines and secretory factors increased (Fig. 1e). For stereo-seq data, a total of 467,017 bins at bin50 resolution (50 × 50 DNA nanoballs (DNBs)) were analyzed from infected hearts and mock control from 1 to 7 dpi for clustering, revealing distinct time-dependent transcriptional changes (Supplementary Fig. 2a, b and Supplementary Tables 3, 4).

Myocarditis-affected regions, delineated by areas of inflammation as evidenced by hematoxylin and eosin (H&E) staining, exhibited an initial scattered distribution at 1–2 dpi and subsequently coalesced, nearly occupying the entire cardiac tissue by 6–7 dpi (Fig. 1f, Supplementary Fig. 1d and Supplementary Fig. 2c, d). Infection response scores defined by the upregulated genes at 7 dpi versus control also increased (Fig. 1g). Furthermore, quantitative spatial area analysis consistently demonstrated a pronounced enlargement of the affected myocarditic area in conjunction with a proportional reduction in ventricle region (Fig. 1h). By integrating snRNA-seq and stereo-seq, our study delineated the myocarditic region’s cellular landscape, identifying macrophages, T cells, neutrophils, and B cells as the main cell populations (Fig. 1i and Supplementary Fig. 2e), with macrophages exhibiting the highest prevalence (Fig. 1i–j and Supplementary Fig. 2e).

In summary, we successfully built a spatiotemporal transcriptional landscape of FM mice and identified the heterogeneous cell-type distribution.

### Spatially restricted gene expression patterns in myocarditis tissues

**Fig. 2:**
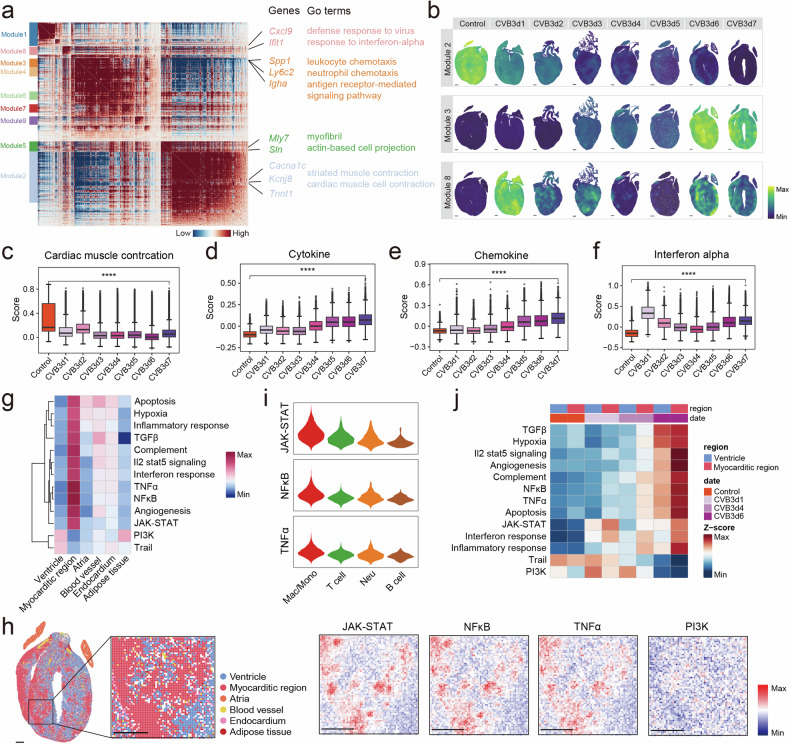
Distinct transcriptional patterns of the hearts during FM progression. **a** Heatmap showing the genes with significant spatial autocorrelation grouped into different modules based on pairwise spatial correlations on cardiac tissue sections. Selected genes and their corresponding GO terms related to representative gene modules are highlighted on the right side. **b** The expression of module genes on cardiac tissue sections from control and CVB3-infected mice from 1 to 7 dpi. Boxplots of cardiac muscle contraction score (**c**), cytokine score (**d**), chemokine score (**e**) and interferon alpha score (**f**) of control and CVB3-infected mice from 1 to 7 dpi. **g** Heatmap of pathway enrichment on different spatial structures. **h** The pathway activation status on the typical cardiac tissue section of 7 dpi. Scale bar: 500 μm. **i** The activation status of typical inflammatory pathways in different types of immune cells. **j** The activation of pathways in the ventricle and myocarditic region at different time points

To examine the transcriptional dynamics during FM progression, we examined the gene expression patterns in FM mouse hearts based on stereo-seq data. A total of 9 modules were identified (Fig. 2a and Supplementary Table 5). Several modules exhibited distinct spatial patterns, such as that module 5 was stably enriched in the atrial myocardium (Supplementary Fig. 3a). Loss of contractile function in cardiomyocytes was suggested by the considerable decrease in module 2 expression, which was enriched in striated muscle contraction function (Fig. 2a, b). This was consistent with the observed decline in cardiac function during FM progression. Module 3 became more prevalent as the disease progressed and was primarily enriched in inflammation-related pathways (Fig. 2a, b), indicating the gradual activation of the immune responses during CVB3 infection. At 1 dpi, module 8, which was mostly enriched in defense response to virus and response to interferon alpha (IFN-α) pathways, exhibited notable enrichment (Fig. 2a, b). To confirm these results, we calculated the gene scores based on snRNA-seq data. The inflammation and apoptosis scores also showed a time-dependent elevation in the hearts of FM mice, whereas the cardiac muscle contraction score declined and interferon alpha score peaked at 1 dpi (Fig. 2c–f and Supplementary Table 6). Apart from that, Mfuzz revealed 4 clusters with similar time-dependent expression patterns (Supplementary Fig. 3b). Genes in cluster 2 (543 genes) showing elevated expression over time. These genes were strongly enriched in the biological processes linked to the ability of the immune system to fight infection and inflammation (Supplementary Fig. 3b–c). Cluster 4 (615 genes) exhibited a decline in the expression of genes associated with cardiac contractile function over time (Supplementary Fig. 3b–c). Cluster 3 (364 genes) was highly expressed at 1 dpi and was associated with viral response processes (Supplementary Fig. 3b–c). To determine the source of the enhanced IFN-α release at 1 dpi, we calculated the proportion of immune cells around CVB3 positive cells (Method) and discovered that neutrophils were the most abundant. Besides, neutrophils had the highest IFN-α score at 1 dpi, indicating that they were the first responders to the disease (Supplementary Fig. 3d–f). Depleting neutrophils at day 0 rather than day 4 reduced the death rate and cardiac inflammation, improved cardiac function of mice with FM (Supplementary Fig. 3g–j). This was in accordance with our previous finding that neutrophils played a significant role in the early stage of FM.^21^

Next, we explored gene expression patterns in different spatial areas. PROGENy analysis indicated that the ventricular region was mainly associated with the phosphoinositide 3-kinase (PI3K) and tumor necrosis factor (TNF)-related apoptosis-inducing ligand (TRAIL) pathways (Fig. 2g). PI3K pathways support the contractile function of the healthy myocardium.^22^ Meanwhile, as TRAIL is a ligand for death receptor 5, it has been observed that its altered expression influences the protective role of apoptosis in suppressing inflammation, indicating a risk of cardiomyocyte damage in FM.^23^ The myocarditic region showed activation of inflammatory response-associated pathways, including TNF-α and nuclear factor-κB (NF-κB) pathways (Fig. 2g–h), with macrophages being significantly activated (Fig. 2i). Further analysis indicated that these inflammatory pathways were significantly elevated in the later stage of the disease. Intriguingly, the cells of the ventricular regions also showed significant inflammatory activation and hypoxia pathways at 6 dpi of the disease. This indicated that normal cardiomyocytes were also inflamed in the later stage (Fig. 2j).

In summary, integrated snRNA-seq and stereo-seq analysis of FM mouse model identified distinct transcriptional dynamics underlying FM pathogenesis, including time-dependent interferon and immune activation, cardiac functional decline, and predisposition to cardiomyocyte death.

### Mesothelial cells trigger the host innate immune responses

**Fig. 3:**
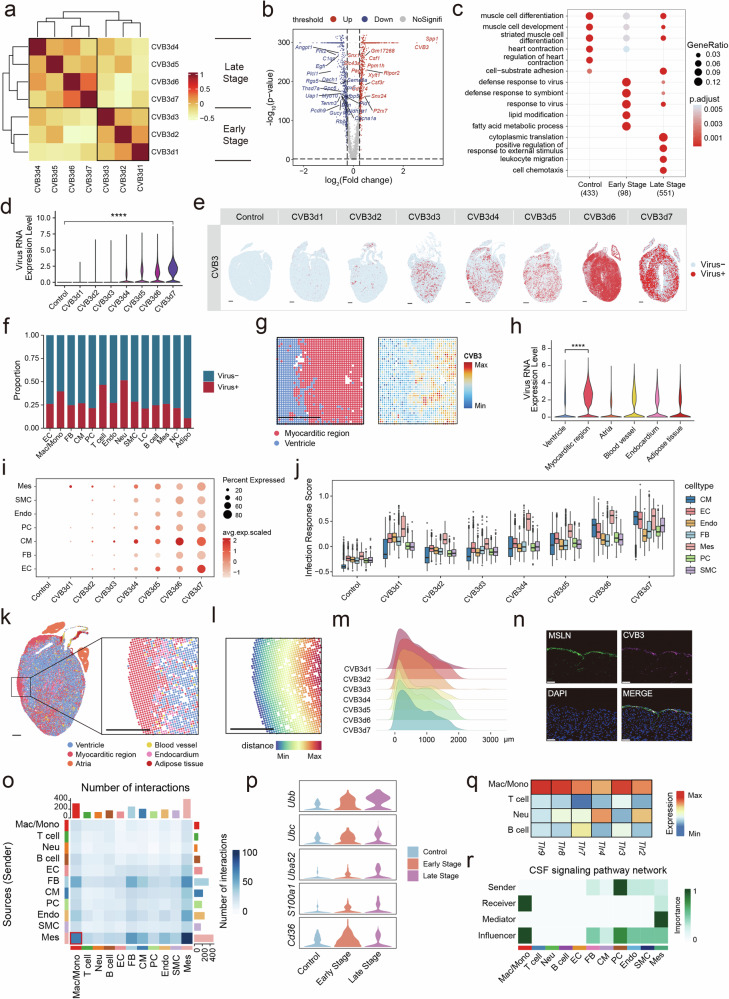
CVB3 infection of cardiac structure cells and immune cells. **a** The stage delineation of disease progression based on Pearson correlation. **b** Volcano plot of regulated genes when compare the cells of early and late stages. **c** Dot plot shows the GO terms of specifically expressed genes in cardiomyocytes of each stage. **d** Violin plot showing the level of CVB3 in cells of control and CVB3-infected mice heart from 1 to 7 dpi in the snRNA-seq data. **e** The level of CVB3 in cardiac tissue sections from control and CVB3-infected mice from 1 to 7 dpi. **f** The proportion of CVB3 positive cells in all cell types in the snRNA-seq data. **g** The typical image of CVB3 mRNA distribution in the cardiac section. Scale bar: 500 μm. **h** Violin plot of CVB3 level in different spatial regions. **i** Bubble plot showing the level of CVB3 in each cell type of control and CVB3-infected mice hearts from 1 to 7 dpi in the snRNA-seq data. **j** Infection response score for cardiac structure cells in snRNA-seq data across control and CVB3 infected hearts from 1 to 7 dpi. **k** Representative spatial image of myocarditic region that distributed near the border region from CVB3-infected mice heart at 4 dpi. Scale bar: 500 μm. **l** Representative spatial image of the distance of myocarditic region to the border region of CVB3-infected mice heart at 4 dpi. Scale bar: 500 μm. **m** The distance of myocarditic region to the border region of CVB3-infected mice hearts from 1 to 7 dpi. **n** Immunofluorescence staining of MLSN and CVB3 showing infected mesothelial cells at 1 dpi. Scale bar: 50 μm. **o** Heatmap showing the number of signaling interaction among immune cells and structural cells in the early stage. **p** Violin plot showing the expression level of damage-associated molecular pattern genes in mesothelial cells of control and CVB3 infected mice at different stages. **q** Heatmap shows the expression level of toll-like receptors in major immure cell types. **r** Heatmap shows the interaction of the CSF signaling pathway among cardiac immune cells and structure cells. EC endothelial cell, Mac/mono macrophage/monocyte, FB fibroblast, CM cardiomyocyte, PC pericyte, T T cell, Endo endocardial cells, Neu neutrophil, SMC smooth muscle cell, LC lymphatic cell, B B cell, Mes mesothelial cell, NC neuron cell, Adipo adipocyte

To better characterize the FM developmental process, we identified the early (1-3 dpi) and late stages (4-7 dpi) of the disease using Pearson’s correlation analysis (Fig. 3a). Compared to the early stage, immune gene expression was more drastic, with significant activation of immune-related pathways in the late stage (Fig. 3b, c). Meanwhile, virus response-related pathways were significantly enriched in the early stage (Fig. 3c). As expected, the level of CVB3 showed a time-dependent increase, indicating viral replication after CVB3 injection in the snRNA-seq data (Fig. 3d). To evaluate the viral invasion status at a spatial resolution, we examined the stereo-seq data of the infected hearts from 1 to 7 dpi. CVB3-positive cells were originally dispersed in the early stage, and then dramatically proliferated over the entire heart at the late stage, particularly at 6 and 7 dpi (Fig. 3e). Next, we examined the CVB3 distribution in each cell type. The results indicated that CVB3 was detected in all cell types (Fig. 3f; Supplementary Fig. 4a, b). Remarkably, the spatial distribution of CVB3 RNA matched to the myocardial area, with the myocarditic region exhibiting the highest viral expression (Fig. 3g, h). This demonstrates that in the late stage of disease, there is an urgent need for cells with robust immunological functions to engulf and process viruses.

To investigate the pathogenesis initiation, we measured the level of CVB3 in structural cells. The results indicated that mesothelial cells had the highest level of CVB3 at 1 dpi (Fig. 3i; Supplementary Fig. 4a, b). Mesothelial cells also showed the highest infection response score during disease progression (Fig. 3j). To explore the heterogeneity of infected cells in more detail, we reclustered cardiomyocytes, endothelial cells and fibroblasts using snRNA-seq data. We found three inflamed subclusters (CM2, EC2, and FB2) with higher CVB3 level and infection response score in cardiomyocytes, endothelial cells and fibroblasts, respectively. (Supplementary Fig. 5a–l). However, mesothelial cells showed higher level of CVB3 and infection response score compared with the three inflamed subclusters at the early stage (Supplementary Fig. 5m, n). As mesothelial cells are mostly distributed in the pericardium of the heart, the pericardium may have been the first area of the heart to be infected with CVB3. This is consistent with the observation that inflammation first appears near the pericardium and then spreads to the entire heart (Fig. 3k). To ascertain this, we calculated the distance of myocarditic region to the border of the heart (Fig. 3l and Supplementary Fig. 5o). The results indicated that the distribution of myocarditic region was closer to the outer border of the heart during disease progression (Fig. 3m). The immunofluorescence staining experiment also validated the presence of viral RNA in mesothelial cells at 1 dpi (Fig. 3n).

CellChat analysis revealed close intercellular communications between macrophages and mesothelial cells in the early stage (Fig. 3o). This indicated the potential role of mesothelial cells in activating macrophages through *C3*/*C3ar1* and *C3/(Itgam+Itgb2)* (Supplementary Fig. 6a). Besides, mesothelial cells expressed several damage-associated molecular patterns after infection, such as *Ubb*, which fueled the inflammatory responses (Fig. 3p and Supplementary Fig. 6b). We then examined the expression of pattern recognition receptors in immune cells and found that they were mainly expressed on macrophages (Fig. 3q), and mainly expressed in the early stage of the disease ((Supplementary Fig. 6c). Mesothelial cells are also important mediators of the colony stimulating factor (CSF) signaling pathways that support the growth of macrophages (Fig. 3r and Supplementary Fig. 6d, e).

Collectively, these results indicate that mesothelial cells are the primary targets of CVB3 in the heart. After infection, mesothelial cells could activate and nourish macrophages to boost infection.

### Macrophages exhibit strong pro-inflammatory activity and recruit cytotoxic T cell

**Fig. 4:**
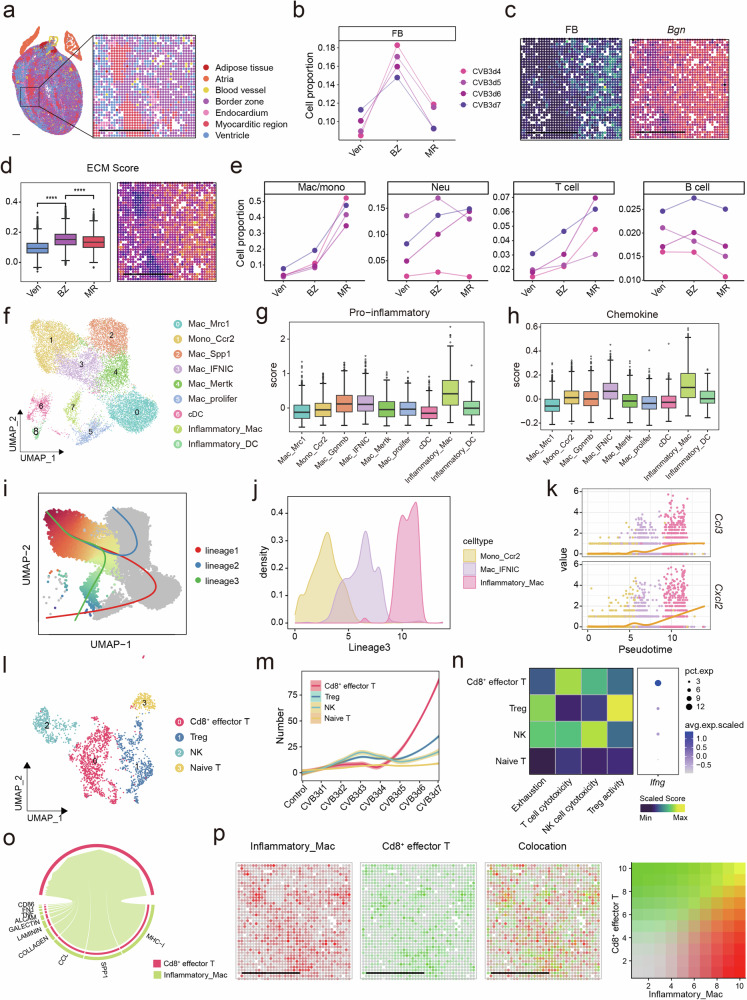
Macrophages chemotaxis activate cytotoxic T cells. **a** Spatial transcriptomic map of cardiac tissue section from CVB3-infected mice at 6 dpi colored by spot clusters representing transcriptionally distinct tissue regions. Scale bar: 500 μm. **b** Changes in average predicted fibroblast proportion across the infected ventricle. **c** Spatial distribution of fibroblast (left) and the expression of *Bgn* on the spatial section (right). **d** Box plot of ECM score among ventricle region, border zone and myocarditic region (left) and ECM score on the spatial section (right). **e** Changes in average predicted cell type proportions across the infected ventricle. **f** UMAP embedding of macrophages colored by manually annotated clusters. Box plot of pro-inflammatory (**g**) and chemokine (**h**) scores among different macrophage subclusters. Scores were calculated by AddModuleScore function of the Seurat package. **i** UMAP embedding shows the developmental trajectory of macrophage subclusters. **j** The densities of Mono_Ccr2, Mac_IFNIC and Inflammatory_Mac along the developmental trajectory. **k** The expression of *Ccl3* and *Cxcl2* along the developmental trajectory. **l** UMAP embedding of T cells colored by manually annotated clusters. **m** The number of T cell subpopulations during disease progression. **n** The functional gene sets and *Ifng* expression level in T cell subpopulations. **o** The interaction pathways between Inflammatory_Mac and Cd8^+^ effector T. **p** The spatial colocation of Inflammatory_Mac and Cd8^+^ effector T cells. Red means the abundance of Inflammatory_Mac, green means the abundance of Cd8^+^ effector T, yellow means the colocation of two cell types. Scale bar: 500 μm. Ven ventricle, BZ border zone, MR myocarditic region

Significant focal accumulation of immune infiltration was observed in the late stage of the disease. To better characterize the features of FM, we defined the border zone that tightly surrounded the myocarditic region in the heart sections in the late stage (Fig. 4a and Supplementary Fig. 7a). As anticipated, the myocarditic region was primarily enriched in processes related to inflammation, whereas the ventricle region was mostly linked to heart contraction function. However, several collagen-associated pathways were found to be enriched in the border zone (Supplementary Fig. 7b), which was consistent with the highest abundance of fibroblasts in this zone (Fig. 4b). The border zone enrichment pattern of fibroblasts and their representative gene expression were also observed in spatial sections (Fig. 4c and Supplementary Fig. 7c). The extracellular matrix (ECM) score was also elevated in the border zone, and the expression of fibroblast genes was positively correlated with ECM score (Fig. 4d and Supplementary Fig. 7d). Cell interaction analysis indicated that myeloid cells released several inflammatory mediators to induce the accumulation and activation of fibroblasts (Supplementary Fig. 7e).

From the ventricle region to the border zone and then to the myocarditic region, several pathways such as TNF-α, NF-κB, Janus kinase–signal transducer and activator of transcription (JAK-STAT), and hypoxia pathways were all elevated (Supplementary Fig. 7f), indicating an increased inflammatory response in the myocarditic region. We then analyzed the immune cell changes from the border zone of the ventricle region to the myocarditic region. Macrophages and T cells were significantly enriched in the myocarditic region (Fig. 4e), indicating their important roles in the cardiac inflammatory response. Therefore, we analyzed these two types of immune cells in more detail.

Here, we identified distinct myeloid cell subgroups at different stages of FM using unbiased clustering. Nine distinct macrophage and dendritic cell populations were identified based on the expression of the relative marker genes (Fig. 4f, Supplementary Fig. 8a–d, and Supplementary Table 7). At 1-2 dpi Mac_Mrc1 is the predominant subtype of macrophage, while the proportions of Inflammatory_Mac, Mac_IFNIC, Mac_Gpnmb, Mono_Ccr2 and Mac_Mertk gradually increase as disease progress (Supplementary Fig. 8e–g). Among these cells, Inflammatory_Mac has the highest pro-inflammatory and cytokine score, and is enriched in several inflammatory-associated pathways (Fig. 4g, h), indicating that this cell type plays a significant role in the inflammatory process. Mono_Ccr2 was migrated from peripheral blood. As named as Mac_IFNIC, this cell population highly expressed IFN-α, and was mainly associated with IFN-related pathways (Supplementary Fig. 8h). To explore the origin of Inflammatory_Mac, we performed trajectory analysis on all macrophage subpopulations and identified 3 distinct trajectories (Fig. 4i). The trajectory 3 indicated that Inflammatory_Mac was developed from Mono_Ccr2 along Mac_IFNIC (Fig. 4j, k and Supplementary Fig. 8i). Trajectory analysis using Monocle2 acquired similar results (Supplementary Fig. 8j). These results indicate that monocytes from the peripheral blood develop into Mac_IFNIC after activation by CVB3. After that, these cells develop into Inflammatory_Mac, and exert various pro-inflammatory effects.

Next, we analyzed T cells with high granularity. Four T cell and NK cell subpopulations were annotated (Fig. 4l, Supplementary Fig. 9a, b and Supplementary Table 8), and Cd8^+^ effector T cells were the most abundant in the late stage (Fig. 4m and Supplementary Fig. 9c). Spatial data also indicated the high abundance of Cd8^+^ effector T cells at 7 dpi (Supplementary Fig. 9d). In addition, Cd8^+^ effector T cells showed the strongest T cell cytotoxicity with high *Fasl* and *Gzmb* expression (Fig. 4n and Supplementary Fig. 9e, f). The pathway enrichment of Cd8^+^ effector T cells indicated that they were associated with virus response pathways and released interferon gamma (IFN-γ) (Fig. 4n; Supplementary Fig. 9g). Cd8^+^ effector T cells also highly expressed chemokines of the CCR family, such as *Ccr2* and *Ccr5*, indicating their recruitment to the inflammatory sites (Supplementary Fig. 9h).

Cell communication analysis revealed that macrophages strongly interacted with the Cd8^+^ effector T cells (Supplementary Fig. 10a–c), and Inflammatory_Mac chemotaxis activated Cd8^+^ effector T cells by MHC-I and CCL molecules (Fig. 4o and Supplementary Fig. 10d–h). Inflammatory macrophages also showed strong spatial colocalization with Cd8^+^ effector T cells (Fig. 4p).

Collectively, the macrophages and T cells were significantly enriched in the myocarditic region. After migration to the heart, peripheral monocytes developed into Mac_IFNIC and Inflammatory_Mac. Inflammatory_Mac chemotaxis activated Cd8^+^ effector T cells, causing tissue damage.

### IFN-γ released by Cd8^+^ effector T cell induces cardiomyocyte death by Spi1

**Fig. 5:**
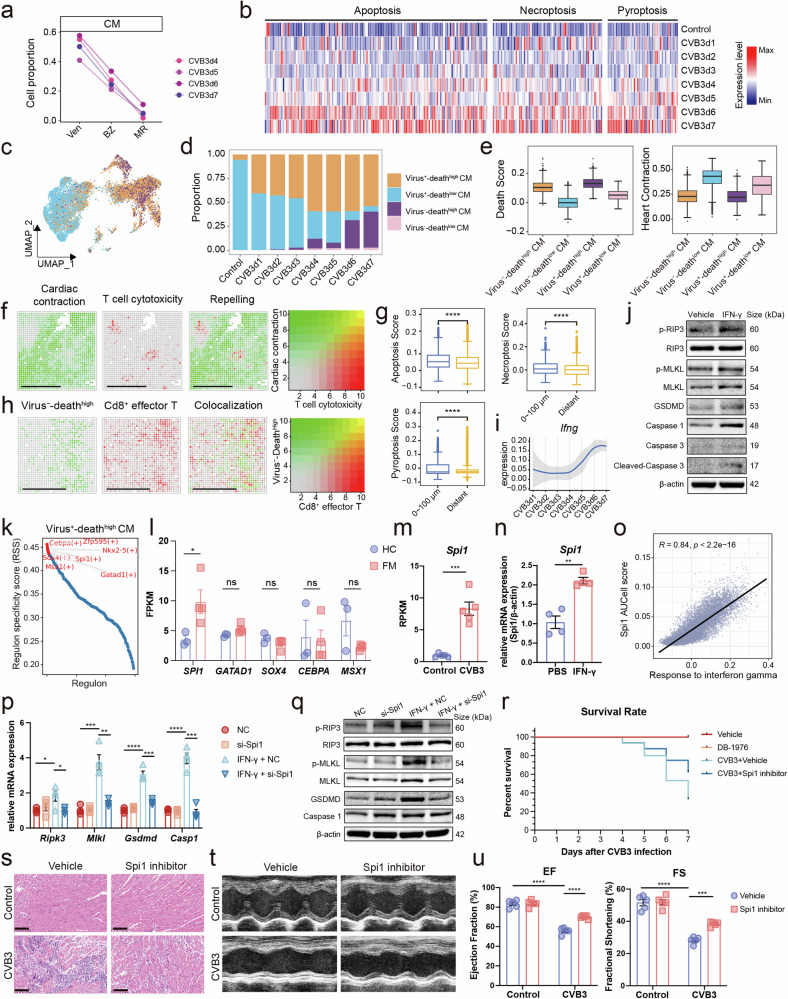
Cd8^+^ effector T cells induced the damage of cardiomyocytes. **a** Changes in average predicted proportion of cardiomyocytes across the infected ventricle in the late stage. **b** The expression of apoptosis, necroptosis, and pyroptosis genes during disease progression in the snRNA-seq data. **c** UMAP embedding of cardiomyocytes of the snRNA-seq data colored by manually annotated clusters. **d** Cell proportion changes of cardiomyocytes at different time points. **e** Box plots of death score and heart contraction score among different cardiomyocyte subclusters. Scores were calculated by AddModuleScore function of the Seurat package. **f** The spatial colocation of contraction genes and T cell cytotoxicity genes. Green means the expression of cardiac contraction genes, red means the expression of cardiac T cell cytotoxicity genes, and yellow means the co-expression status. Scale bar: 500 μm. **g** Box plots of apoptosis, pyroptosis, and necroptosis scores between cardiomyocytes that are near to Cd8^+^ effector T cells and those distant to Cd8^+^ effector T cells. **h** The spatial colocation of virus^-^-death^+^ cells and Cd8^+^ effector T cells. Green means the abundance of virus^-^-death^+^ CMs, red means the abundance of Cd8^+^ effector T cells, and yellow means the colacation of the two cell types. Scale bar: 500 μm. **i** Expression trend of *Ifng* in T cells along different infection times (fitted with loess). The line shadow showing the 95% confidence interval. **j** Western blot images of representative death genes expression in the vehicle and IFN-γ treated mouse primary cardiomyocytes. **k** The top regulons that activated in virus^-^-death^high^ CMs. **l** The expression of key transcriptional factors in the heart of patients with FM (n = 3 in HC group, n = 4 in FM group, data are represented as mean ± SEM). **m** The expression of *Spi1* in the heart of FM mice and control (n = 5 per group, data are represented as mean ± SEM). **n** The expression of *Spi1* in IFN-γ treated mouse primary cardiomyocytes (n = 4 per group, data are represented as mean ± SEM). **o** Correlation between response to interferon gamma score and Spi1 AUCell score of cardiomyocytes**. p** The mRNA level changes of death genes in IFN-γ and si-Spi1 treated mouse primary cardiomyocytes (n = 4 per group, data are represented as mean ± SEM)**. q** Protein level changes of death genes in IFN-γ and si-Spi1 treated mouse primary cardiomyocytes. Survival rate (**r**), H&E staining images (**s**), representative echocardiography images (**t**) and cardiac functions (ejection fraction and fraction shortening) (**u**) changes of FM and Spi1 inhibitor-treated mice. (*n* = 5 per group, data are represented as mean ± SEM). Scale bar: 500 μm. Ven ventricle, BZ border zone, MR myocarditic region, HC healthy control, FM fulminant myocarditis

Cardiomyocytes (CMs) are the most important structural and functional cells of the heart. They are significantly lost during FM progression (Fig. 1f). From the ventricle region to the border zone and then to the myocarditic region, the proportion of CMs decreased significantly (Fig. 5a), indicating damage to the cardiomyocytes at the site of inflammation. As the disease progressed, the expression of the death genes by CMs increased significantly in the snRNA-seq data (Fig. 5b). Apart to CVB3-positive cardiomyocytes, CVB3-negative cardiomyocytes also had higher death scores than control (Supplementary Fig. 11a), which highlights its important role in immune-mediated tissue damage. We further identified four cardiomyocyte subpopulations in the snRNA-seq data according to the level of CVB3 and death score (Fig. 5c and Supplementary Fig. 11b, c). The majority CMs in the healthy heart were virus^-^-death^low^ CMs with normal contraction function (Fig. 5d, e and Supplementary Fig. 11d), only a small portion of virus^-^-death^high^ CMs existed in the healthy heart (Fig. 5d). As the disease progressed, the number of virus^-^-death^low^ CMs significantly decreased, whereas two cell populations with high death gene expression, virus^-^-death^high^ and virus^+^-death^high^ CMs, increased significantly, which expressed higher inflammation and death genes (Fig. 5d, e and Supplementary Fig. 11e–j). Intriguingly, the proportion of virus^-^-death^high^ CMs was much more than that of virus^+^-death^high^ CMs (Fig. 5d). These results indicated that immune-mediated damage may cause drastic harm to CMs. As described previously, Cd8^+^ effector T cells exhibited high cytotoxicity and expressed high level of IFN-γ (Fig. 4n and Supplementary Fig. 9e, f), which may induce damage of cardiomyocytes. Spatial co-localization analysis indicated that the expression of cardiac contraction genes and T cell cytotoxicity genes was strongly repelled (Fig. 5f and Supplementary Fig. 11k). In addition, CMs that were close to Cd8^+^ effector T cells had higher apoptosis, necroptosis, and pyroptosis scores than those that were distant from Cd8^+^ effector T cells (Fig. 5g). Moreover, the specific genes of virus^-^-death^high^ CMs had a strong co-localization with Cd8^+^ effector T cell, highlighted the importance of T cell-mediated damage of CMs (Fig. 5h). As described before, Cd8^+^ effector T cell specifically expressed *Ifng* (Fig. 4n), and the expression of *Ifng* in T cells increased during the time of infection (Fig. 5i and Supplementary Fig. 12a). Correlation analysis indicated the death score of CMs had a high correlation with interferon gamma response rather than the expression of *Gzmb* (Supplementary Fig. 12b, c). Depleting Cd8^+^ effector T cell using CD8α neutralizing antibody in the late stage decreased the death rate of mice with FM, improved cardiac function and reduced cardiac inflammatory infiltration (Supplementary Fig. 12d–h), the level of IFN-γ in the heart also decreased (Supplementary Fig. 12i). To explore the role of IFN-γ in the death of cardiomyocytes, we treated primary mouse cardiomyocytes with recombinant IFN-γ and discovered that IFN-γ could strongly induce the expression of death genes in CMs (Fig. 5j and Supplementary Fig. 13a). Moreover, IFN-γ antibody treatment could significantly reduce the death rate of FM mice (Supplementary Fig. 12j), improve cardiac function (Supplementary Fig. 12k, l) and alleviate cardiac inflammatory infiltration (Supplementary Fig. 12m).

Next, we explored the mechanism behind Cd8^+^ effector T cell released IFN-γ mediated damage of CMs. Regulon modulation analysis indicated that virus^-^-death^low^ healthy cells were modulated by transcriptional factors such as Hand2 (Supplementary Fig. 12n, o), which have been reported to maintain the normal function of cardiomyocytes.^24^ However, virus^-^-death^high^ cells are mainly regulated by transcriptional factors such as Spi1 and Gatad1 (Fig. 5k and Supplementary Fig. 12p). To increase the clinical relevance of these results, we performed bulk RNA sequencing on 4 heart samples of FM patients and 3 healthy controls. The results indicated that most of the up-regulated genes in the myocarditic region of FM mice also existed in the heart samples of FM patients (Supplementary Fig. 12q). Intriguingly, SPI1 is the only transcriptional factor that elevated in the hearts of FM patients among transcriptional factors that modulate virus^-^-death^high^ CMs (Fig. 5l). Besides, the expression of SPI1 was positively correlated with the expression of several death genes (Supplementary Fig. 12r). So, we mainly focused on *Spi1* in following research. In the bulk RNAseq data of the heart from FM mice that we reported previously,^25^ *Spi1* also significantly elevated (Fig. 5m). Besides, IFN-γ could directly induce the expression of *Spi1* in mouse primary cardiomyocytes (Fig. 5n). Moreover, the activity of Spi1 had a strong correlation with the genes in response to IFN-γ (Fig. 5o). To explore the relationship of *Spi1* in IFN-γ mediated cardiomyocyte death, we isolated the mouse primary cardiomyocytes and treated with IFN-γ and si-Spi1. The results indicated that silencing Spi1 could rescue IFN-γ mediated cardiomyocyte death both in mRNA (Fig. 5p) and protein level (Fig. 5q and Supplementary Fig. 13b). Apart from that, we treated FM mice with Spi1 inhibitor, the death rate of FM mice significantly reduced, the immune infiltration decreased, and the heart function of FM mice significantly improved (Fig. 5r–u).

These results indicate that Cd8^+^ effector T cells could release IFN-γ, which induced the death of CMs by modulating *Spi1*.

### Intravenous immunoglobulin (IVIG) treatment reduces inflammation in FM

**Fig. 6:**
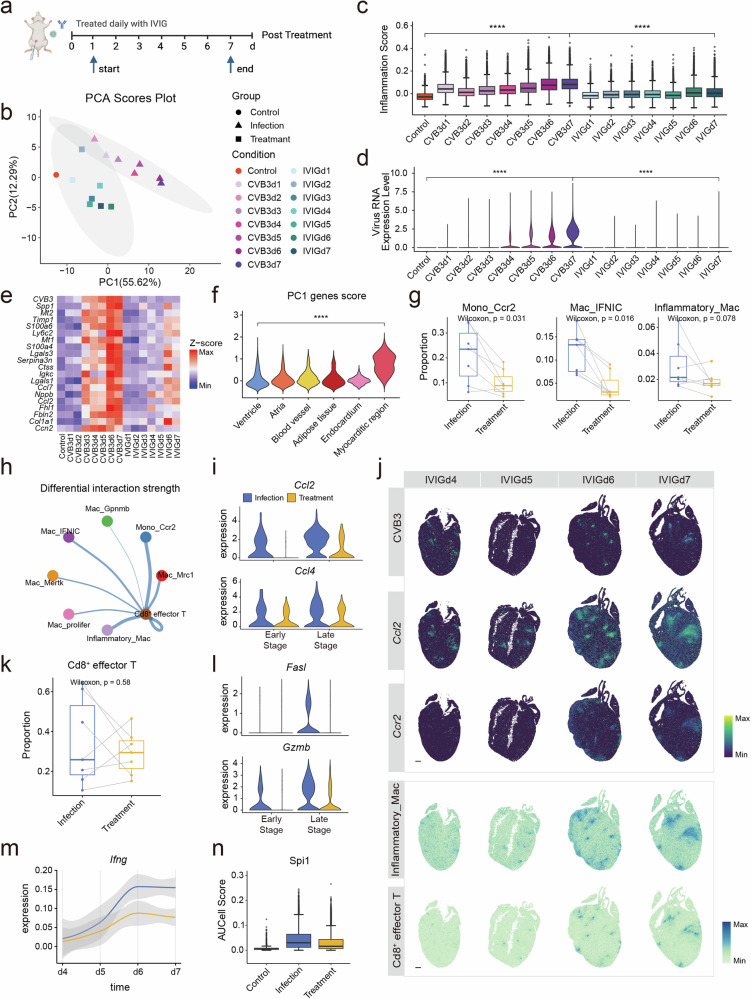
The treatment effects of IVIG on FM mice. **a** Schematic diagram of the workflow for drug administration. **b** PCA analysis of control, infection and treatment samples from 1 to 7dpi. **c** The level of inflammation score in control, infection and treatment samples from 1 to 7dpi. **d** The level of CVB3 in control, infection and treatment samples from 1 to 7dpi. **e** Heatmap showing the expression level of PC1 genes. **f** Violin plot shows the expression of PC1 genes in different spatial areas. **g** The proportion of Mono_Ccr2, Mac_IFNIC and Inflammatory_Mac of infection and treatment groups. **h** Differential strength of cellular interactions among cardiac macrophages and T cells in infection and treatment groups. The blue line indicates a decrease in the treatment group. **i** The expression of chemokines (*Ccl2* and *Ccl4*) by Inflammatory_Mac in infection and treatment groups at different stages. **j** The level of CVB3, the expression of *Ccl2* and *Ccr2*, and the distribution of Inflammatory_Mac and Cd8^+^ effector T cells on cardiac sections in treatment group in the late stage. **k** The proportion of Cd8^+^ effector T cells in infection and treatment groups. **l** The expression of cytotoxic molecules (*Fasl* and *Gzmb*) by Cd8^+^ effector T cells in infection and treatment groups at different stages. **m** Expression trend of *Ifng* in T cells of infection and treatment groups along different infection time (fitted with loess). The line shadow showing the 95% confidence interval. **n** The AUCell score of Spi1 in control, infection and treatment groups. PC principle component

As overactivated immune responses are involved in the pathogenesis of FM, we explored the therapeutic effects of IVIG, a well-known immunomodulatory drug, on FM. We performed single-nucleus RNA-seq combined with stereo-seq on the cardiac sections extracted from control and IVIG treated CVB3-infected A/J mice at 1–7 dpi (Fig. 6a). The administration of IVIG reduced death rate of FM mice from nearly 70% to 0% (Supplementary Fig. 14a). Principal component analysis also indicated the samples after treatment were distributed closer to the control sample than to the CVB3 samples at the transcriptional level (Fig. 6b). Both inflammation score and virus RNA reduced after IVIG treatment (Fig. 6c, d). Spatially, most altered genes were expressed in the myocarditic region (Fig. 6e, f) and the area of the myocarditic region decreased after treatment (Supplementary Fig. 14b, c), indicating the strong immunomodulatory effect of IVIG. IVIG also decreased the infiltration of several types of immune cells, especially pro-inflammatory macrophages (Fig. 6g and Supplementary Fig. 14d). As macrophages play key roles in chemotaxis and activation of Cd8^+^ effector T cells, the reduced number of inflammatory macrophages results in decreased communication between macrophages and Cd8^+^ effector T cells (Fig. 6h). The reduced release of chemokines from macrophages further decreased the number of Cd8^+^ effector T cells (Fig. 6i–k). Consequently, the number of cytotoxic molecules as well as *Ifng* expressed by T cells was also decreased in treatment group (Fig. 6l, m), thereby decreasing the activity of Spi1 in cardiomyocytes (Fig. 6n). The death of cardiomyocytes also decreased (Supplementary Fig. 14e–g).

These results demonstrate the potent immunomodulatory effects of IVIG, highlighting its therapeutic potential for FM.

## Discussion

FM is a life-threatening cardiac inflammatory disease with an exceptionally high mortality rate. Despite its severity, the pathogenesis of FM remains poorly understood, hindering the development of effective diagnostic and therapeutic strategies.^26^ Comprehensive investigation of the mechanisms underlying the overactivated inflammatory response in FM is critical for advancing clinical diagnosis, treatment, and prevention. High-throughput technologies such as integrated high-throughput snRNA-seq and spatial transcriptomics have proven valuable for studying cardiac microenvironments in various heart diseases.^27,28^ Many studies have explored the immune mechanisms in different types of myocarditis.^18,19^ For instance, in autoimmune myocarditis, hypoxia-inducible factor 1α (Hif1α) modulates immune responses by influencing macrophage clusters and T-helper 17 cells.^17^ In ICI-induced myocarditis, pathogenic CD8^+^ T cells expand in both the blood and heart,^29,30^ while *Cxcl9*^+^ and *Cxcl10*^+^ macrophages play an essential role in disease progression via IFN-γ signaling.^31^ However, the chronological progression of FM and its underlying immune mechanisms remain largely unknown.

In this study, we utilized ST-seq and snRNA-seq to dissect the spatial and cellular heterogeneity in CVB3-induced FM in an A/J mouse model. We identified the patterns of changes in the heart during disease progression, investigated the affected cell types, and assessed the cellular heterogeneity and spatial distribution of both immune cells and cardiac structure cells. Furthermore, we generated snRNA-seq and spatial transcriptome datasets at seven time points with and without IVIG treatment. Our data provide detailed insights into the chronology of the molecular events leading to CVB3-induced FM. Notably, we discovered that mesothelial cells were the first infected cells to develop the disease. According to the disease nature, the heart is the only organ that suffers direct virus attack. The absence of viral reads in peripheral blood mononuclear cells indicated that the infected cardiac structural cells were the culprit to chemotaxis and activated peripheral macrophages and the virus-positive immune cells were infected only after they enter to local heart. As the cells envelop the heart, the mesothelial cells give rise to multiple cardiac cell lineages during embryonic development and are essential for myocardial growth and repair.^32–34^ Infection of mesothelial cells results in epicarditis of the heart, which is an early manifestation of FM. In this study, we discovered that mesothelial cells had the highest proportion of CVB3-positive cells and the highest infection response score at 1 dpi. Besides, the inflammation always tends to appear near the epicardium region of the heart. These indicated that the infected mesothelial cells may play an important role in chemotaxis and activating cardiac macrophages at the very early stage of the disease. Additionally, infected mesothelial cells release several damage-associated molecular patterns activating the pro-inflammatory macrophages at the early stage of the disease. However, it cannot rule out the role of the virus and other infected structural and immune cells in activating macrophages in the late stage during the disease progression. Macrophages play a significant role in the development of myocarditis.^35^ Specifically, tissue-resident macrophages exert protective effects against cardiovascular diseases, whereas mononuclear macrophages derived from peripheral blood often exert pro-inflammatory and tissue-destructive effects.^36,37^ In autoimmune myocarditis, elevated expression of hypoxia-inducible factor 1α modulates macrophages to induce tissue damage.^17^ Cxcl9/Cxcl10^+^ macrophages interact with T cells via IFN-γ and CXCR3 signalling pathways and cause myocardial cell damage in immune checkpoint inhibitor-induced myocarditis.^31^ In this study, we identified a distinct developmental trajectory. The pro-inflammatory monocytes that migrated from the peripheral blood developed into Mac_IFNIC and then differentiated into Inflammatory_mac. The Inflammatory_Mac has the highest pro-inflammatory and chemotactic activity and activates Cd8^+^ effector T cells by releasing chemokines of the CCL family and elevating the expression levels of MHC molecules. Cd8^+^ effector T cells subsequently release IFN-γ and cytotoxic molecules, such as granzyme B, to induce the cardiomyocyte death, thereby reducing cardiac function.

IVIG therapy, which involves immunoglobulins derived from the plasma of healthy donors, is widely used to treat a variety of conditions, including immunodeficiencies, autoimmune diseases, and infection-related acute illnesses.^38^ Beyond providing passive immunity against viruses, IVIG exhibits potent immunomodulatory effects, including binding cytokines and variable regions of other antibodies. It has been employed to manage viral infection such as severe acute respiratory syndrome coronavirus, Middle East respiratory syndrome coronavirus, and COVID-19.^39^ Although IVIG has become a routine treatment for myocarditis based on case reports and series,^40^ its efficacy in FM remains contentious. While some case reports demonstrate that IVIG effectively treats FM,^41–43^ a large retrospective study of 2,423 acute myocarditis and 648 FM cases found no significant reduction in in-hospital mortality with IVIG treatment.^44^ Our study revealed that IVIG not only reduced viral load but also exerted profound immunomodulatory effects, significantly attenuating cardiac inflammation in FM mice. Specifically, IVIG decreased the number of Inflammatory_Mac cells and Cd8^+^ effector T cells, thereby reducing their interactions. Importantly, we discovered a novel mechanism by which IVIG suppresses IFN-γ release from T cells, thereby reducing IFN-γ-induced *Spi1* expression and subsequent cardiomyocyte death. These findings highlight the significant therapeutic potential of IVIG in managing FM by targeting the hyperinflammatory state and preserving cardiac function.

Despite the valuable insights provided by our study, certain limitations should be noted. First, our focus on the heart as the primary organ of interest precluded an examination of other organs potentially affected by FM. Second, reliance on a single animal model may limit the generalizability of our findings. Moreover, specific cell subsets and conclusions drawn from cell-state transition and cellular interaction analyses also require further experimental validation. Future studies employing advanced techniques such as lineage tracing could provide deeper insights into immune cell development and its role in FM. Additionally, investigating the inflammatory microenvironment and its impact on cardiac damage using human samples will be crucial for translating these findings to clinical settings.

In conclusion, our study presents a comprehensive spatiotemporal landscape of FM, offering unique insights into its pathophysiology. By integrating snRNA-seq and spatial transcriptomics, we identified previously unknown functional pathways and spatial cell interactions in FM. These findings provide a detailed understanding of the disease’s pathogenic mechanisms and lay the foundation for developing novel therapeutic strategies.

## Materials and methods

### Animal studies

Six-week-old male A/J mice were obtained from Gem Pharmatech (Nanjing, China). To build model of FM, A/J mice (*n* = 20) were intraperitoneally injected with 1 × 10^4^ plaque-forming units of CVB3, as previously described.^21^ Control mice (*n* = 5) were injected with the same dosage of phosphate-buffered saline. The survival events and body weight changes of mice were recorded daily until death or day 7. At the end of the experiment, all animals were anesthetized via an intraperitoneal injection of a mixture of xylazine (5 mg/kg) and ketamine (80 mg/kg) before euthanasia. The cardiac functional changes of mice were recorded by echocardiography using the Vevo1100 high-resolution imaging system with 30-MHz high-frequency scan head (Visual Sonics, Canada). In the IVIG-treated group, the IVIG was intraperitoneally administered at a dose of 1 mg/kg daily from days 0 to 7. For drug treatment experiments, six-week-old male AJ mice were randomly divided into 4 groups: phosphate-buffered saline (PBS) group, SPI1 inhibitor DB1976 dihydrochloride (2.5 mg/kg) group or interferon (IFN) gamma antibody (5 mg/kg) group, with or without CVB3 injection. After a seven-day interval post-administration of CVB3, the mice underwent echocardiographic evaluation and were subsequently sacrificed. Spi1 inhibitor DB1976 dihydrochloride (Cat. HY-135797A) and Anti-Mouse IFN gamma Antibody (H22) (Cat. HY-P99136) were purchased from MedChemExpress (New Jersey, USA) and were given after CVB3 injection. To deplete CD8^+^ effector T cells, 100 μg of anti-CD8α antibody (Cat. BE0090, BioXcell, USA) was injected i.p on day 4. The same dosage of rat IgG (Cat. BE0090, BioXcell, USA) was used as control. Neutrophil depletion was induced with anti-Ly6G mAb clone 1A8 (Cat. BE0075, BioXcell, USA) injected at 0.25 mg i.p on day 0 or day 4. The same dosage of rat IgG (Cat. BE0089, BioXcell, USA) was used as control. The samples were collected after euthanasia.

### Histological analysis

Cardiac tissue samples were embedded in paraffin and preserved in 4% paraformaldehyde. Then, tissue slices of 4-μm thickness were cut and stained for immunohistochemical, immunofluorescence, and hematoxylin and eosin staining (H&E). The probe used to detect CVB3 RNA in immunofluorescence staining was synthesized by Servicebio (Wuhan, China). All antibodies used in this study are listed in Supplementary Table 9.

### Sample preparation and sequencing of single-nucleus RNA-seq

Single-nucleus mRNA libraries were constructed for sequencing using the Chromium Next GEM Single Cell 3 GEM Library and Gel Bead Kit v3.1 (#1000121). The samples were sequenced using Illumina NovaSeq 6000. Reads from snRNA-seq were aligned to a 10X pre-built mouse reference (refdata-gex-mm10-2020-A) and collapsed into UMI counts using the 10x Genomics Cell Ranger software (version 7.0.0) with default parameters. We converted count matrices into Seurat objects using the R package Seurat (v4.0.2).^45^ Potential doublets in dataset were removed with DoubletFinder (v3.076),^46^ in which the parameter was 0.076 for assuming doublet formation rate. For quantity filtering, dead cells and low-expression genes were identified respectively by high mitochrondial percentage (>10%) and low gene/feature counts per cell (<5) and filtered out. The cells that expressed less than 300 unique genes were removed. Then, NormalizeData function with scale.factor = 10,000 was performed on all cells to eliminate the influence of technical factors such as sequencing depth. We used Harmony^47^ to integrate the data of different batches to remove batch effects. FindVariableFeatures function in Seurat was used for feature selection, and 5000 highly variable genes were selected for downstream analysis. The integrated cells were constructed in the top 50-dimensional PCA space to construct a KNN graph and performed Louvain clustering with the FindClusters function, and visualized the data using UMAP dimensionality reduction. We annotated the obtained cell clusters by combining the reported cell markers and the cluster markers identified under the default parameters of FindAllMarkers function. For the cell subpopulations, we perform the above steps under the selected main cell cluster.

### Stereo-sequencing (stereo-seq)

Stereo-seq experiment, library preparation, and sequencing were performed as described in an established pipeline.^48^ After RIN value inspection of the tissue blocks, they were cut into 100–200-μm thick sections embedded in the OCT compound. Total RNA was extracted using a RNeasy Mini Kit (Qiagen). Only samples with RIN ≥ 7 can meet the conditions for spatial transcriptome research. Tissue permeabilization was tested with varying durations (3, 6, 12, 18, 24 min) on the Stereo-seq Chip P Slide using 10 µm tissue sections, with a positive control of mouse heart tissue. The optimal permeation time was detected through imaging with a fluorescence microscope with the strongest fluorescence and no dispersion. When the permeation time was set to 18 minutes, the details were clear, the signal was uniform, and the brightness was maximum. Therefore, the optimal permeation time was determined to be 18 minutes. Then we proceed to the formal Stereo-seq experiment. Briefly, following cryosection, tissue sections are mounted on Stereo-seq chips, incubated at 37 °C, and fixed with 20 °C methanol. The chips underwent ssDNA staining and imaging with a Nikon Ti-7 Eclipse microscope. Tissue permeabilization was performed with 0.1% pepsin in HCl buffer, followed by washing with SSC buffer containing RNAase Inhibitor. Captured mRNA was reverse transcribed at 42 °C. Post in situ reverse transcription, the chips were treated with tissue removal buffer and then washed. cDNA was released from the chips using a cDNA Release Mix at 55 °C, followed by fragmentation, amplification, and purification to produce a DNBs library. The DNBs were sequenced on the MGI DNBSEQ-T1 platform.

### Stereo-seq raw data processing (Alignment and UMI counting)

The Stereo-seq FASTQ data contains unique identifiers (25 bp CID and 10 bp MID) in the first read and cDNA sequences in the second read. We used the SAW workflow (https://github.com/BGIResearch/SAW) to process this data, which involves several key steps, including mRNA spatial location restoration, filtering, mRNA genome alignment, gene region annotation, MID (Molecule Identity) correction, expression matrix generation, and tissue region extraction. Two core tools in SAW accomplish these steps. We utilized the SAW software’s mapping tool to align read1’s CIDs with the Stereo-seq chip’s coordinates, allowing a single base mismatch. This resulted in “Valid CID Reads,” which were then supplemented with coordinate data. The subsequent “Clean Reads” were obtained by filtering out MIDs with polyA sequences and low-quality bases (N or more than two bases with a score below 10), as well as mRNA with polyA. The Clean reads were aligned to the mouse reference genome (GRCm39/mm10) with the extra CVB3 genome from National Center for Biotechnology Information (https://www.ncbi.nlm.nih.gov/) with the accession number GenBank:NC_038307.1, using STAR (v2.5.3),^49^ and the number of reads aligned to regions such as exons, introns, and intergenic regions were counted according to the gene annotation files. Using Bam2Gem (https://github.com/BGIResearch/handleBam), the corresponding relationships between the unique mapping reads aligned to the reference genome and the genes were determined, and the expression levels of all genes were calculated according to MID correction. Through quantification of gene expression, the final output was the expression matrix of all genes detected in the tissue section, which was stored in a GEM format file. The bin1 represents one spot with 220 nm diameter in section, and bin n represents combined bins in an N × N square area.

### Image processing and cell segmentation

First, the total number of genes under a single DNB was transformed into an image and registered with the single-stranded DNA staining image using ImageJ^50^ software. The Python package StereoCell^51^ was used to perform nuclear segmentation. The GMM implemented in StereoCell was used to fit the molecular distribution under a cell-nucleus mask. The probability of extracellular molecules with a fitting range (100 × 100 pixels) was then calculated according to the GMM, and the pixels outside the mask were resegmented to obtain the complete cell boundary. Unique molecular identifiers from all DNBs in the same putative single cell (referred to as a cellbin) were aggregated to generate a gene expression matrix for downstream analysis.

### Spatial quantification of immune cells in the virus-positive spots

Considering the artifact noise introduced by stereo-seq experiments or sequencing, to ensure the authenticity of CVB3 virus presence within the spots, only spots with a minimum of two CVB3-related UMI were considered virus-positive. Then, we calculated the proportions of immune cells within 0–25, 25–50, 50–75, 75–100, and >100 μm radius around the virus-positive spots based on the results of deconvolution.

### SnRNA-seq data processing

Raw sequencing data processing (alignment, filtering, and UMI counting) was performed using the CellRanger toolkit v2.1.0. cDNA reads were mapped to the modified reference genome described above. The aligned reads were then filtered to generate gene-cell matrices for downstream analysis. Count matrices were converted into Seurat objects using the R package Seurat (v4.0.2).^45^ Potential doublets in the dataset were removed using DoubletFinder (v3.076),^46^ in which the parameter was set to 0.076 to assume a doublet formation rate. Additionally, dead cells and low-expression genes were identified by high mitochondrial percentage (>10%) and low gene/feature counts per cell (<5) and filtered out. Cells expressing fewer than 300 unique genes were excluded from the study. Then, the NormalizeData function with a scale factor of 10,000 was performed on all cells to eliminate the influence of technical factors, such as sequencing depth. We used Harmony^47^ to integrate data from different batches to remove batch effects. The FindVariableFeatures function in Seurat was used for feature selection, and 5,000 highly variable genes were selected for downstream analysis. The integrated cells were constructed in the top 50-dimensional principal component analysis space to construct a KNN graph, Louvain clustering was performed with the FindClusters function, and the data were visualized using uniform manifold approximation and projection (UMAP) dimensionality reduction. The obtained cell clusters were annotated by combining the reported cell markers and cluster markers identified using the default parameters of the FindAllMarkers function. For the cell subpopulations, we performed the above steps using the selected main cell clusters.

### Differential gene and Gene Ontology (GO) enrichment analysis

The MAST^52^ method wrapped in the Seurat^45^ (v4.0.2) FindMarkers function was used to detect differentially expressed genes between conditions. The differentially expressed genes with min.pct >0.1, logfc.threshold >0.5 and the adjusted *p* value < 0.05 were selected for downstream analysis. The enrichGO function of ClusterProfiler package^53^ (v4.0) was used for GO enrichment analysis, and the GO terms of adjusted *p* < 0.05 (Benjamini–Hochberg method) was selected.

### Gene regulatory network

We constructed a gene regulatory network for cardiomyocytes based on the pyscenic CLI pipeline.^54^ Specifically, GRNBoost2 was used to construct an adjacency matrix between transcript factors and target genes in the filtered raw count matrix. The pruned candidate regulons were identified based on DNA motif analysis using RcisTarget. The activity score of each regulon in each cell was measured using AUCell. Jensen–Shannon divergence was used to calculate the regulon specificity score (RSS). Finally, we sorted the regulons by RSS, selected the regulons of interest, and used the ClusterProfiler package^53^ (v4.0) for the functional enrichment of their target genes.

### Signature gene set scoring

Gene signature scores were calculated to evaluate the expression levels of individual cells or bins using the AddModuleScore function of the Seurat^45^ R package with the default parameters. Gene sets for GO biological processes and HALLMARK was obtained from the Molecular Signatures Database (https://www.gsea-msigdb.org/gsea/msigdb) using the msigdbr package.^55^ All signature genes are listed in Supplementary Table 6.

### Dynamic expression pattern clustering and functional analysis

Mfuzz R package^56^ was used to identify the time-dependent transcriptional programs in the single-cell transcriptome data during infection. This tool is based on a fuzzy c-means algorithm to examine the temporal trends of gene expression and cluster genes exhibiting analogous expression profiles. Optimal values of parameters c and m were derived for this analysis. GO functional enrichment analysis of gene clusters was conducted to investigate their functions and understand the changes in biological processes.

### Cell-cell communication analysis

To assess the communication networks of different cell populations, cell-cell communications from snRNA-seq data were analyzed using CellChat^57^ (v1.4.0). First, we set Secreted Signaling as a predetermined ligand database to infer the probability of cell-cell communication. After identifying the significant ligand-receptor pairs, we used the computeCommunProbPathway function to infer the interactions between cells at the pathway level. Visualization of specific pathways was performed using Circos plots with the netVisual_circle function and heatmap plots with the netVisual_heatmap function.

### Trajectory analysis

Inferred pseudo-time trajectories of monocytes and macrophages were analyzed using the R package, slingshot^58^ and monocle.^59^ For monocle pipeline, the gene expression matrix with 2,000 high variable genes was reduced using the DDRTree algorithm wrapped in reduceDimension function. For slingshot pipeline, UMAP coordinates were used to visualize the cell lineage trajectories for each cell type, and the cluster “Mono_Ccr2” was set as a possible starting point. A cluster-based minimum spanning tree (MST) was used to stably identify the global lineage structure, and the getCurves function was used to fit the smooth branching curves to these lineages.

### Pathway and cell subtype activity inference analysis using spatial data

The Python package decoupleR (v1.12.1)^60^ was used to calculate the pathway activity score for each bin50. For signaling pathways, target genes from the comprehensive resource PROGENy^61^ and msigdb databases were used. The top 30 genes upregulated in each cell subtype were selected as the signature genes. The multivariate linear model (mlm) method implemented in the run_mlm function was used to obtain pathway and cell subtype activity scores.

### Cell type deconvolution

To analyze cell distribution, we used Cell2location (v0.1.3),^62^ a Bayesian model that can resolve fine-grained cell types in spatial transcriptomic, to integrate snRNA-seq and stereo-seq data. Following the official tutorial (https://cell2location.readthedocs.io/en/latest/), low-expression genes in snRNA-seq were identified and filtered, including 1) genes detected in less than five cells. 2) genes detected in at least 5% of the total cell number will be included. 3) genes with average expression in non-zero cells are above 1.12. We used the negative binomial regression to model the snRNA-seq gene expression matrix of different samples. The estimated expression in each cell type was exported with num_samples = 100 and batch_size = 2500. We then fitted the cell2location model with the hyperparameters N_cells_per_location = 4 (bin50)/1 (cellbin) (estimated by the cell area), detection_alpha = 20 (control the regularization). Finally, the q05 cell abundance (5% quantile of the posterior distribution, representing the value of cell abundance that the model has high confidence in) was used for downstream analysis. For a cell bin considered as a single cell, spots were defined as specific cell types with the highest abundance. For bin50 considered as a mixture of a few cells, the cell-type proportions for each spot were calculated using abundance estimations. Finally, the deconvolution results were added to the Seurat object metadata.

### Identification of spatially correlated modules

The Python package hotspot^63^ (v 0.9.0) was used to identify the spatial gene modules based on spatial location. We used 2,000 highly variable genes as inputs and selected 300 neighbors to build a KNN graph using the Bernoulli method to model the gene expression data at each bin50. To determine the genes with spatial variation, the compute_local_correlation function was used to calculate the local correlation and assign it to different gene modules using the create_modules function with a minimal gene threshold of 30 and fdr threshold of 0.05.

### RNA sequencing and data analysis

RNA sequencing was conducted as previously described.^2^ Total RNA was extracted using TRIzol Reagent (Invitrogen, Cat. No. 15596026) following the method established by Chomczynski et al.^3^ After RNA extraction, residual DNA was removed by DNase I treatment. The RNA quality was assessed by measuring the A260/A280 ratio using a NanoDrop OneC spectrophotometer (Thermo Fisher Scientific). The integrity of the RNA samples was further confirmed using 1.5% agarose gel electrophoresis. Final RNA quantification was performed using the Qubit™ RNA Broad Range Assay Kit (Life Technologies, Q10210) on a Qubit™ 3.0 fluorometer. For RNA sequencing, 2 μg of total RNA was used to construct stranded RNA sequencing libraries using the KCTM Stranded mRNA Library Prep Kit for Illumina (Catalog no. DR08402, Wuhan Seq Health Co., Ltd., China) according to the manufacturer’s instructions. Libraries with fragment sizes between 200-500 bp were enriched, quantified, and sequenced on the DNBSEQ-T7 platform (MGI Tech Co., Ltd., China) in paired-end 150 bp mode. Raw sequencing reads were processed using Trimmomatic (v0.36) to remove low-quality reads and adapter contamination. Gene-level read counts were obtained using featureCounts (Subread v1.5.1, Bioconductor), followed by the calculation of RPKM values. DESeq2^64^ R package was used to detect the gene expression difference between healthy and FM patients.

### Neonatal mouse cardiomyocytes isolation

The isolation of neonatal mouse cardiomyocytes (NMCMs) was performed as previously outlined.^65^ Briefly, hearts from 1-day-old C57BL/6 neonatal mice of both sexes were harvested and subjected to digestion using D-Hanks solution (Solarbio, Cat. #H1045), supplemented with trypsin (Gibco, Cat. #27250018) and collagenase type II (Gibco, Cat. #17101-015), with continuous recirculation for 25 minutes. After digestion, the tissue was incubated at 37 °C for 5 minutes to further facilitate digestion, followed by the neutralization of the collagenase with 10 mL of Dulbecco’s Modified Eagle’s Medium (DMEM, Gibco, Cat. #C11965500BT) containing 10% fetal bovine serum (FBS, Gibco, Cat. #10091148). The cell suspension was centrifuged at 1000 rpm for 5 minutes, and the resulting pellet was resuspended in a fresh cell culture medium. Fibroblasts were allowed to adhere to the culture dish for 2 hours at 37 °C in a 5% CO_2_ atmosphere. Subsequently, the cultures were washed twice with DMEM, and the non-adhered cardiomyocytes were collected by centrifugation at 1000 rpm for 5 minutes. The collected cardiomyocytes were then plated and cultured in DMEM supplemented with 10% FBS and 100 U/mL penicillin-streptomycin. NMCMs were cultured for 48 hours before being used for further analysis.

### Cell culture and stimulation

The NMCMs were cultured in DMEM (Gibco, Cat. #C11965500BT) supplemented with 10% fetal bovine serum (FBS, Gibco, Cat. #10091148) and 100 U/mL penicillin-streptomycin (Gibco, Cat. #15140122), and maintained at 37 °C in a humidified incubator with 5% CO_2_. The NMCMs were plated at a density of 1 × 10^6^ or 5 × 10^5^ cells per well in 6-well plates or 12-well plates. For IFN-γ stimulation, NMCMs were incubated with recombinant mouse IFN-γ protein (Abclonal, Cat. # RP01070) at a final concentration of 100 ng/mL for 24 hours.

### siRNA-mediated gene silencing

siRNAs targeting the gene of interest were designed and synthesized by GenePharma (Shanghai, China). To silence the target gene expression, Lipofectamine™ RNAiMAX Transfection Reagent (Thermo Fisher, Cat. #13778150) was used to deliver the siRNAs into NMCMs. Briefly, siRNA was diluted in Opti-MEM (Gibco, Cat. #31985070) and mixed with Lipofectamine™ RNAiMAX reagent at a ratio recommended by the manufacturer. The final siRNA concentration was 100 nM for NMCMs. The siRNA-lipid complex was incubated with the cells for 6 hours. Following transfection, cells were cultured in fresh DMEM containing 10% FBS for another 24 hours, after which they were subjected to stimulation with IFN-γ (100 ng/mL) or left untreated, depending on the experimental conditions.

### RNA isolation and qPCR

Total RNA extracted from mouse primary cardiomyocytes was transcribed into cDNA and analyzed using real-time qPCR. After reverse transcription, cDNA was amplified using specific primers of genes and Taq Pro Universal SYBR qPCR Master Mix (Vazyme, CAT#. Q712-02), by 7900HT Fast Real-Time PCR system (Life Technologies, Carlsbad, CA, USA). All primers used in this study are listed in Supplementary Table 10. Gene expression was then normalized to that of Actb using the comparative CT (ΔΔC_T_) method.

### Protein extraction and Western blot analysis

Western blots were performed as previously described. Briefly, cells and tissues were lysed in RIPA buffer supplemented with protease and phosphatase inhibitors (MCE, Cat. #HY-K0021, and #HY-K0022) to prevent protein degradation and dephosphorylation. The bicinchoninic acid protein assay (Thermo Fisher Scientific, Cat. #23225) was used to determine protein concentration. Equal amounts of protein from each sample were loaded onto a 10% sodium dodecyl sulfate-polyacrylamide gel and separated by electrophoresis. Following electrophoresis, proteins were then transferred onto a polyvinylidene fluoride membrane (Millipore, Cat. #IPVH00010) using a wet transfer system. The membrane was then blocked with 5% nonfat dry milk dissolved in Tris-buffered saline with 0.1% Tween-20 (TBST) at room temperature for 1 hour to minimize non-specific binding. Primary antibodies specific to the target proteins were incubated with the membrane overnight at 4 °C under gentle agitation. After washed in TBST, the membrane was incubated with the appropriate horseradish peroxidase (HRP)-conjugated secondary antibody at room temperature for 1 hour. After thorough washing, protein bands were visualized using enhanced chemiluminescence (ECL) reagents (Thermo Fisher Scientific, Cat. #32106), and the signals were captured and analyzed using the iBright™ 1500 Imaging System (Thermo Fisher Scientific). Band intensities were quantified using ImageJ software (NIH) for semi-quantitative analysis. All antibodies used in this study are listed in Supplementary Table 9.

### Enzyme-linked immunosorbent assay

The level of IFN-γ in the heart of FM mice was measured using a standard enzyme-linked immunosorbent assay kit (Cat#: EMC101g, NEOBIOSCIENCE, China). All measurements were performed following the manufacturer’s instructions.

### Statistical analysis

All the data were represented as mean ± standard deviation. Statistical analysis of snRNA-seq and Stereo-seq was performed using R package and visualized by ggplot2 package. The comparison of differences between two groups was performed by two-sided Mann-Whitney-Wilcoxon with Benjamini-Hochberg correction for multiple comparisons. Pearson correlation analysis for snRNA-seq and stereo-seq data was performed by “cor” function. All *p* values were considered significant at the 0.05 level. The median, 25th (Q1), and 75th (Q3) percentiles are marked in boxplots, whiskers extend to the furthest data point in the range 1.5× (Q3–Q1). In animal studies, the comparisons were analyzed by Student’s t-test between two groups or one-way ANOVA with the Tukey post-test if comparing several groups.

## Supplementary information

Supplementary figures 1-14

Supplementary tables 1-10

## Data Availability

The snRNA-seq and Stereo-seq data supporting the findings of this study have been deposited into the China National GeneBank (CNGB) Sequence Archive (CNSA, accession code: CNP0005824) and Spatial Transcript Omics DataBase (STOmics DB, accession code: STT0000127), respectively.
