# Supplementary material for: Spatiotemporal transcriptomics elucidates the pathogenesis of fulminant viral myocarditis
Source: Signal Transduct Target Ther. 2025 Feb 10;10:59. doi: 10.1038/s41392-025-02143-9 (PMC11808084; doi:10.1038/s41392-025-02143-9)
Supplement: Supplementary file 1 — Supplementary figures 1-14 [file 41392_2025_2143_MOESM1_ESM.pdf]

# Supplementary Materials for

## Spatiotemporal transcriptomics elucidates the pathogenesis of fulminant viral myocarditis

Huihui Li[1]*, Xueting Chen[2,3]*, James Jiqi Wang[1]*, Juan Shen[2,4]*, Kudusi Abuduwufuer[1], Zhao Zhang[2,3], Zhensheng Dong[2], Zheng Wen[1], Jingwei He[5], Silian Chen[2,3], Wanshun Li[5], Chen Chen[1], Fan Li[1], Xiaodong Fang[6,7#], Dao Wen Wang[1#]

Correspondence to: dwwang@tjh.tjmu.edu.cn (D. W); fangxd@genomics.cn (X. F)

**This PDF file includes:**

Figures. S1 to S14

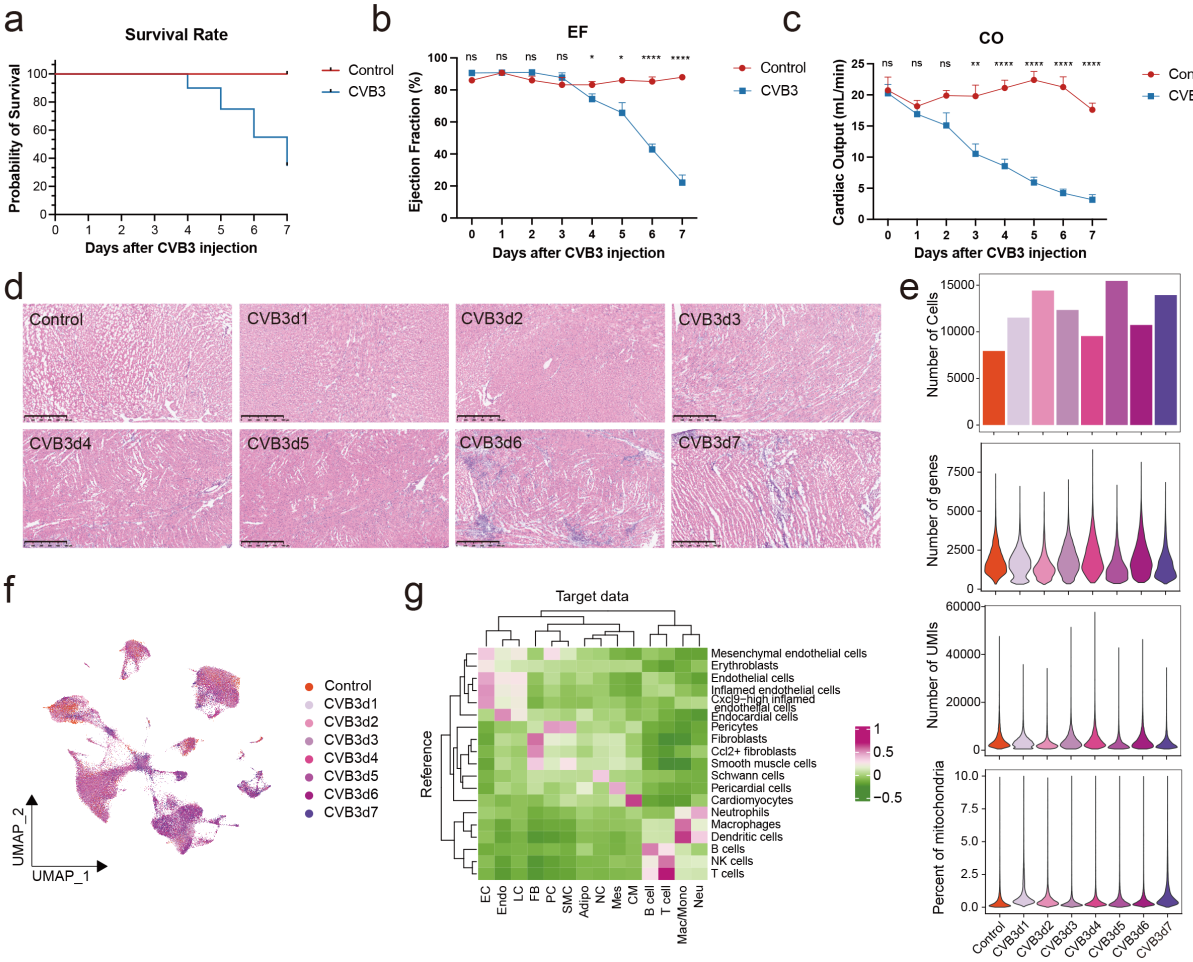


**Figure. S1. The building of FM mice model and single cell transcriptomics of cardiac tissue from CVB3-infected mice.** Survival rate **(a)**, cardiac ejection fraction **(b)** and cardiac output **(c)** of FM mice during disease progression (n=5 per group, data are represented as mean ± SEM). **d,** H&E staining of cardiac tissue sections from control and CVB3-infected mice from 1 to 7 dpi. Scale bar: 500μm. **e,** Number of cells (top), number of unique genes detected per cell (middle top), number of unique transcripts per cell (middle bottom), and percentage of mitochondrial transcripts (bottom) in cardiac snRNA-seq datasets from control and CVB3-infected mice heart from 1 to 7 dpi. **f,** UMAP embedding of all the cells colored by samples. **g,** The heatmap shows the similarity between cells annotated in this article and cells reported by previous research.

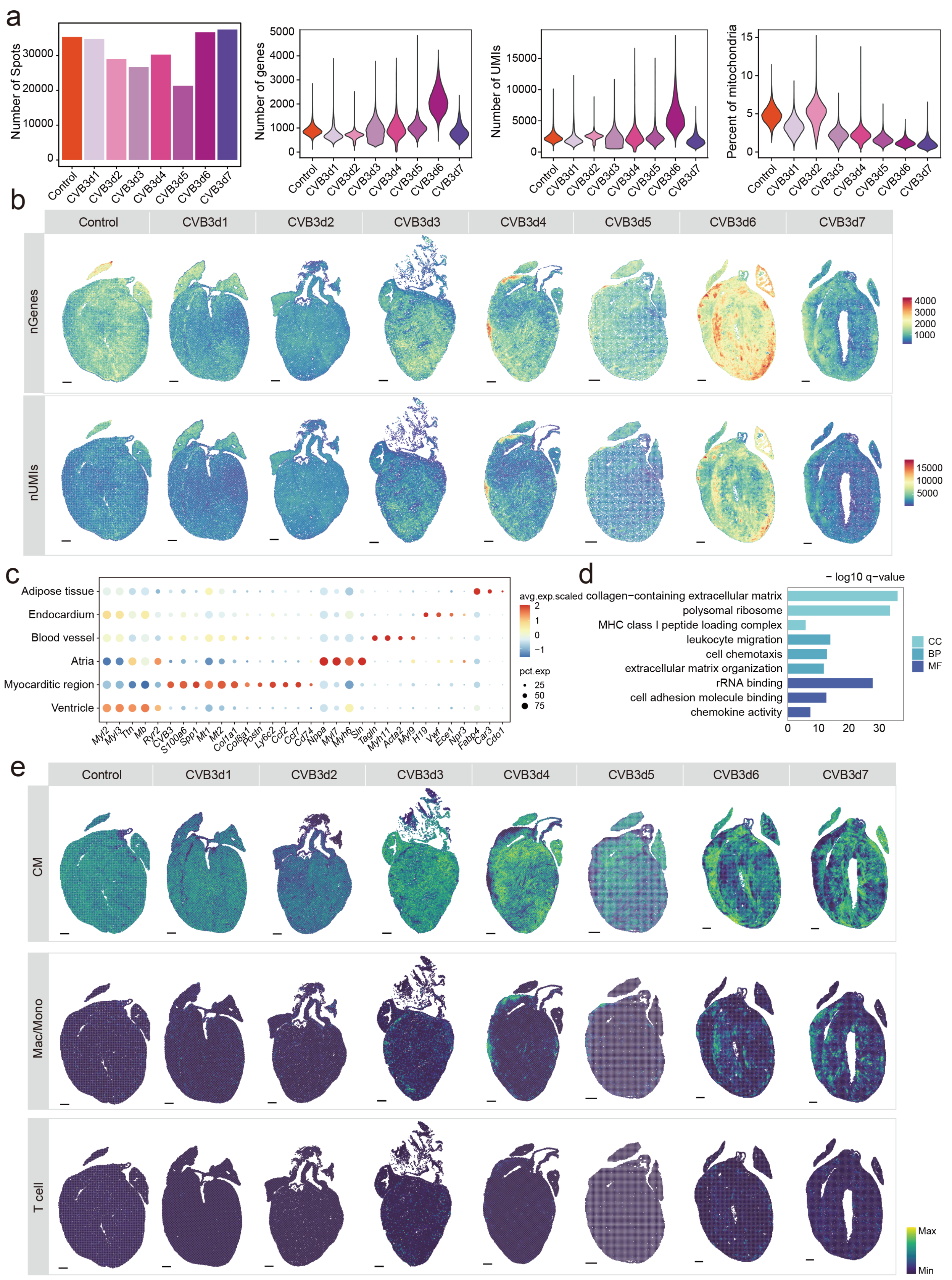
a
Number of Spots
Number of genes
Number of UMIs
Percent of mitochondria
Control
CVB3d1
CVB3d2
CVB3d3
CVB3d4
CVB3d5
CVB3d6
CVB3d7
b
nGenes
nUMIs
c
Adipose tissue
Endocardium
Blood vessel
Atria
Myocarditic region
Ventricle
avg.exp.scaled
pct.exp
Myl2
Myl3
Ttn
Mb
Ryr2
CVB3
S100a6
Spp1
Mt1
Mt2
Col1a1
Col8a1
Postn
Ly6c2
Ccl2
Ccl7
Cd74
Nppa
Myl7
Myh6
Sln
Tagln
Myh11
Acta2
Myl9
H19
Vwf
Ece1
Npr3
Fabp4
Car3
Cdo1
d
− log10 q−value
collagen−containing extracellular matrix
polysomal ribosome
MHC class I peptide loading complex
leukocyte migration
cell chemotaxis
extracellular matrix organization
rRNA binding
cell adhesion molecule binding
chemokine activity
CC
BP
MF
e
CM
Mac/Mono
T cell
Max
Min

**Figure. S2. The spatial transcriptomics of cardiac tissue from CVB3-infected mice. a,** Number of spots (left), number of unique genes detected per spot (middle left), number of unique transcripts per spot (middle right), and percentage of mitochondrial transcripts (right) in cardiac spatial transcriptomic datasets from control and CVB3-infected mice heart from 1 to 7 dpi. **b,** Number of unique genes detected per spot (up) and number of unique transcripts per spot (down) on spatial cardiac section. Scale bar: 500μm. **c,** Dot plot showing the expression levels of characteristic genes in each spatial area. **d,** The gene ontology enrichment of genes that specifically expressed in myocarditic region. **e,** The spatial distribution of CM (up), Mac/mono (middle) and T cells (down) on cardiac sections of control and CVB3-infected mice heart from 1 to 7 dpi. Scale bar: 500μm.

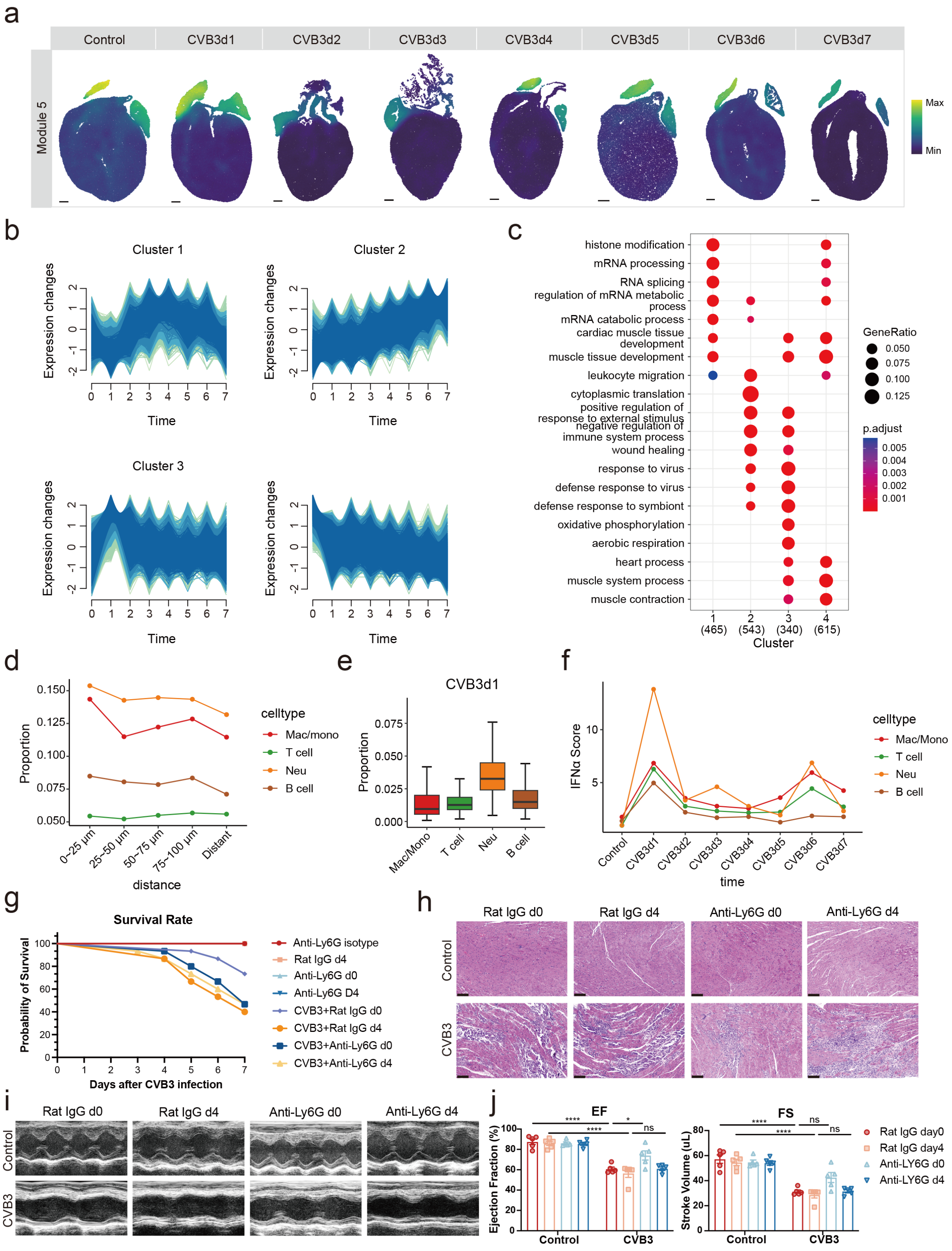
a
Control
CVB3d1
CVB3d2
CVB3d3
CVB3d4
CVB3d5
CVB3d6
CVB3d7
Module 5
Max
Min
b
Cluster 1
Cluster 2
Cluster 3
Expression changes
Time
c
histone modification
mRNA processing
RNA splicing
regulation of mRNA metabolic process
mRNA catabolic process
cardiac muscle tissue development
muscle tissue development
leukocyte migration
cytoplasmic translation
positive regulation of response to external stimulus
negative regulation of immune system process
wound healing
response to virus
defense response to virus
defense response to symbiont
oxidative phosphorylation
aerobic respiration
heart process
muscle system process
muscle contraction
1 (465)
2 (543)
3 (340)
4 (615)
Cluster
GeneRatio
0.050
0.075
0.100
0.125
p.adjust
0.005
0.004
0.003
0.002
0.001
d
Proportion
0~25 μm
25~50 μm
50~75 μm
75~100 μm
Distant
distance
celltype
Mac/mono
T cell
Neu
B cell
e
CVB3d1
Proportion
Mac/Mono
T cell
Neu
B cell
f
IFNα Score
Control
CVB3d1
CVB3d2
CVB3d3
CVB3d4
CVB3d5
CVB3d6
CVB3d7
time
celltype
Mac/Mono
T cell
Neu
B cell
g
Survival Rate
Probability of Survival
Days after CVB3 infection
Anti-Ly6G isotype
Rat IgG d4
Anti-Ly6G d0
Anti-Ly6G D4
CVB3+Rat IgG d0
CVB3+Rat IgG d4
CVB3+Anti-Ly6G d0
CVB3+Anti-Ly6G d4
h
Rat IgG d0
Rat IgG d4
Anti-Ly6G d0
Anti-Ly6G d4
Control
CVB3
i
Rat IgG d0
Rat IgG d4
Anti-Ly6G d0
Anti-Ly6G d4
Control
CVB3
j
EF
FS
Ejection Fraction (%)
Stroke Volume (uL)
Control
CVB3
Rat IgG day0
Rat IgG day4
Anti-LY6G d0
Anti-LY6G d4

**Figure. S3. Transcriptional expression pattern of the heart during disease progression. a,** The expression of module 5 genes on cardiac tissue sections from control and CVB3-infected mice from 1 to 7 dpi. Scale bar: 500μm. **b,** 4 distinct cluster of gene expression pattern during disease progression identified by Mfuzz. **c,** Dot plot showing the representative pathways of four characteristic gene clusters identified by Mfuzz. **d,** Line chart shows the proportion of 4 main types of the immune cells around CVB3 positive cells in different distance at day one after CVB3 infection. **e,** Box plot showing the proportion of immune cells in each Stereo-seq bin50-spot of CVB3d1 heart. **f,** The IFNα score of 4 main types of the cardiac immune cells during disease progression. Mac/mono: macrophage/monocyte; T: T cell; Neu: neutrophil B: B cell. Survival rate **(g)**, H&E staining images **(h)** , representative echocardiography images **(i)** and cardiac functions (ejection fraction and fraction shortening) **(j)** changes of neutrophil depleting mice (n=5 per group, data are represented as mean ± SEM). d0: day0; d4: day4. Scale bar: 100 μm.

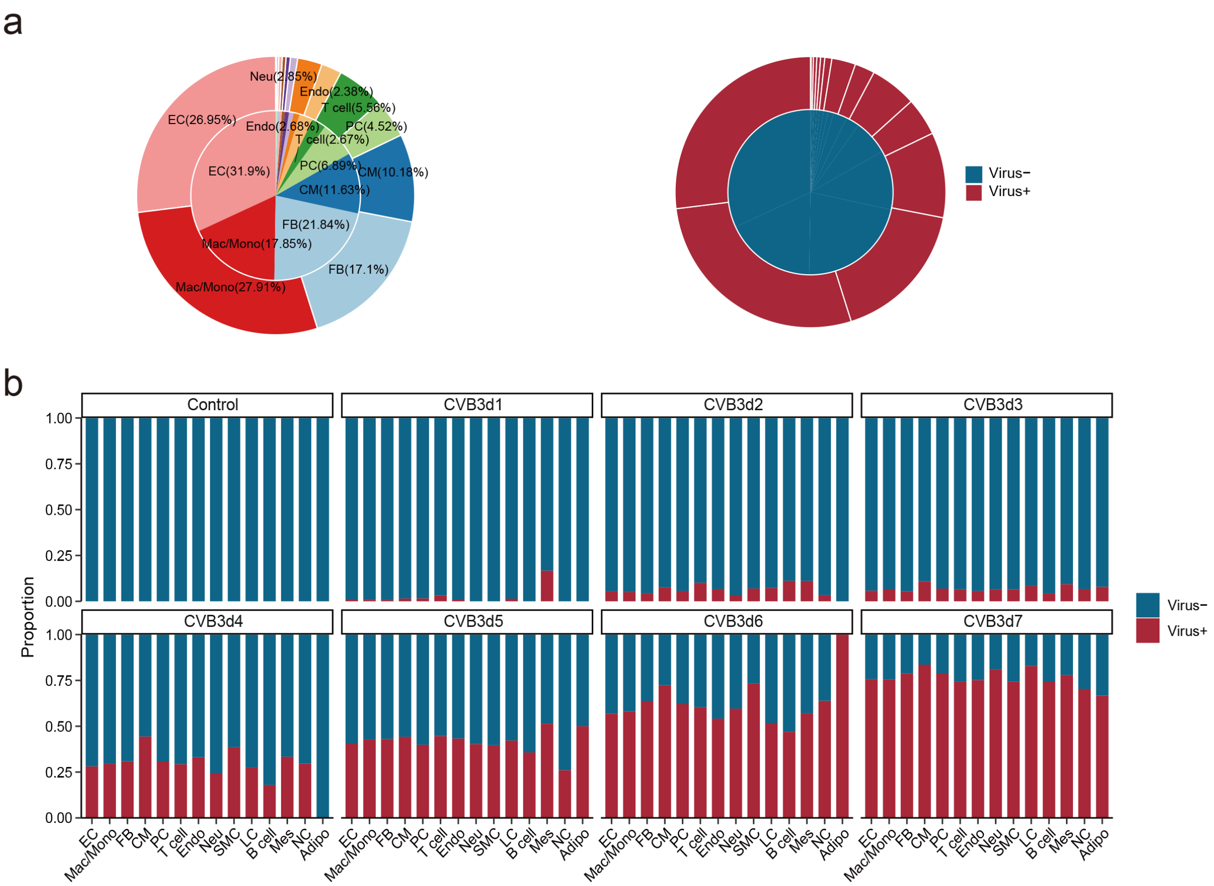


**Figure. S4. The distribution of CVB3 in different cell types. a,** The pie chart shows the proportion of CVB3 positive and CVB3 negative cells. **b,** The proportion of CVB3 positive cells in all cell types of control and CVB3-infected mice from 1 to 7 dpi. EC: endothelial cell; Mac/mono: macrophage/monocyte; FB: fibroblast; CM: cardiomyocyte; PC: pericyte; T: T cell; Endo: endocardial cells; Neu: neutrophil SMC: smooth muscle cell: LC: lymphatic cell; B: B cell; Mes: mesothelial cell; NC: neuron cell; Adipo: adipocyte.

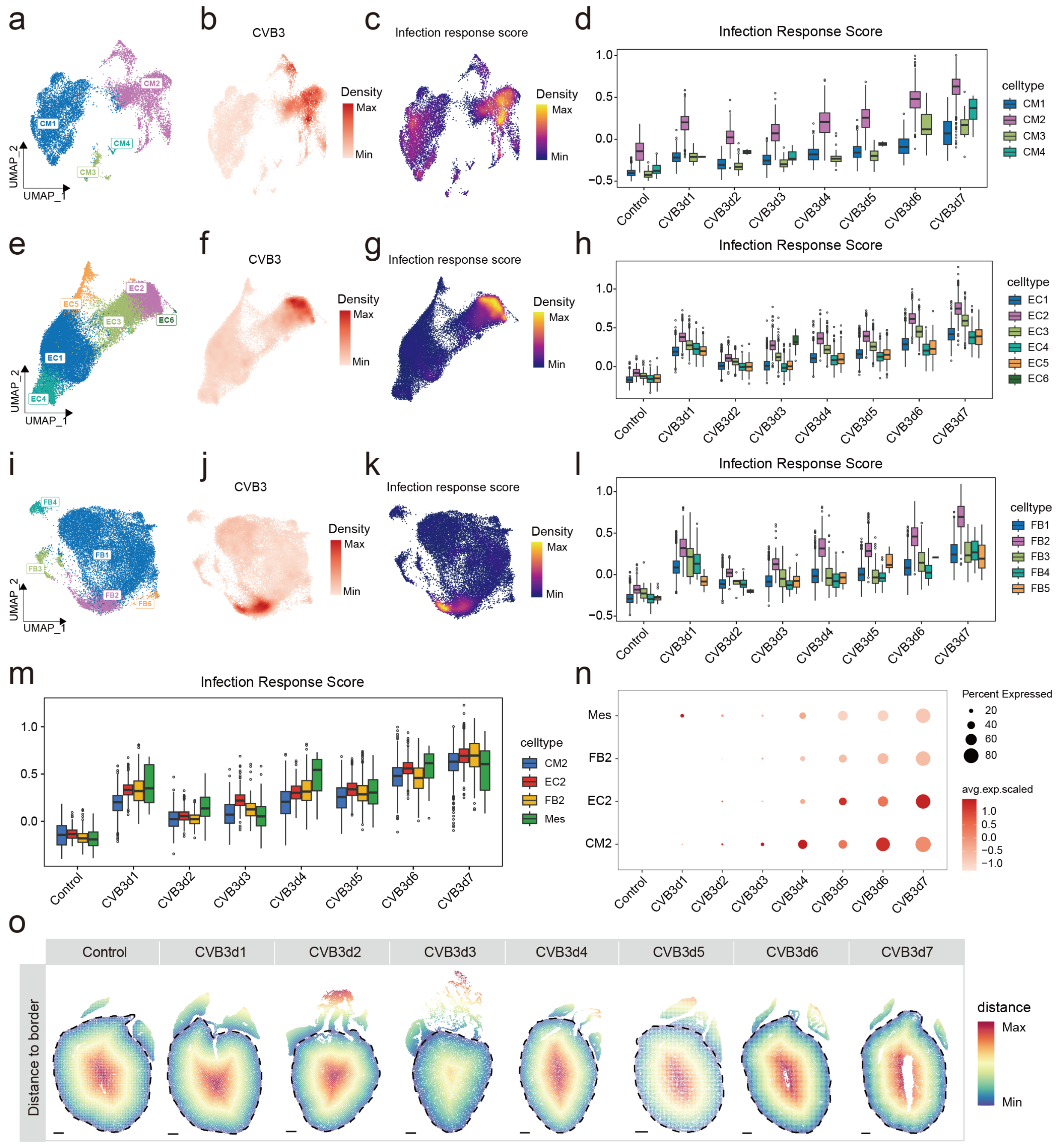


**Figure. S5. The infection response score of mesothelial cells and cardiac structural cell subpopulations. a,** UMAP plot showing four subtypes of CM. **b,** UMAP plot showing the expression density of CVB3 in CM. **c,** UMAP plot showing the expression score of infection response in CM. **d,** Box plot comparing infection response score of four CM subtypes across different infection stage. **e,** UMAP plot showing the six subtypes of EC. **f,** UMAP plot showing the expression density of CVB3 in EC. **g,** UMAP plot showing the expression score of infection response. **h,** Box plot comparing infection response score of six EC subtypes across different infection stage. **i,** UMAP plot showing the six subtypes of EC. **j,** UMAP plot showing the expression density of CVB3 in EC. **k,** UMAP plot showing the expression score of infection

response. **l,** Box plot showing infection response score of four CM subtypes across different infection stage. **m,** Box plot comparing the infection response score of CM2, EC2, FB2 and Mes across different infection stage. **n,** CVB3 expression across CM2, EC2, FB2 and Mes, Bubble size is proportional to the percentage of cells expressing CVB3 and color intensity is proportional to average scaled CVB3 expression. **o,** The distance of myocardial region to the border region of CVB3-infected mice heart from control and CVB3-infected mice heart from 1 to 7 dpi. Scale bar: 500μm. UMAP: uniform manifold approximation and projection; CM: cardiomyocyte; EC: endothelial cell; FB: fibroblast; CVB3: coxsackievirus B3.

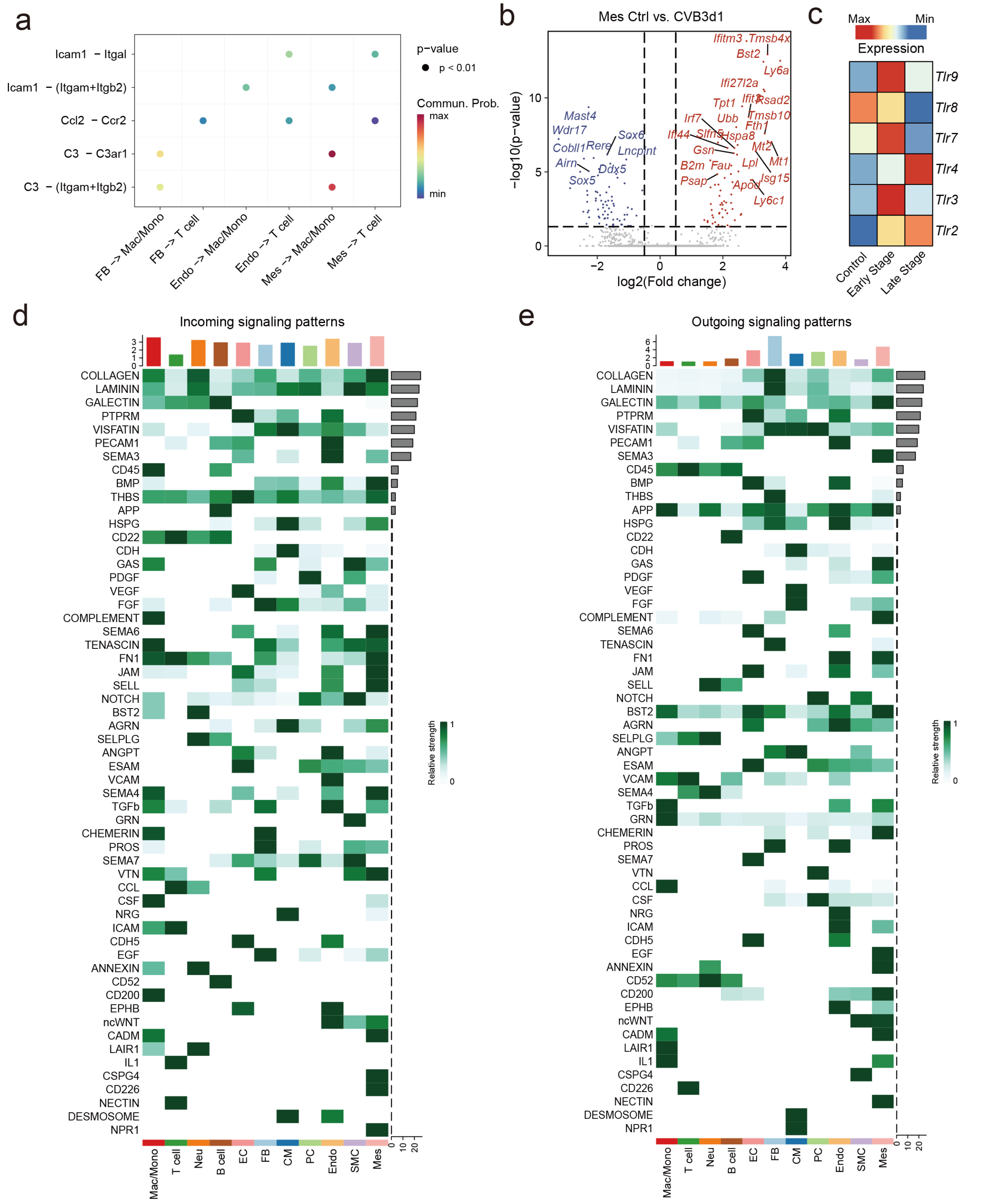


**Figure. S6. Cell interaction among structural cells and immune cells at early stage. a,** Bubble plot showing significant ligand-receptor interaction pairs from structural cells to immune cells. Heatmaps show the signaling strength of each signaling pathway among structural cells and

immune cells. **b,** Volcano plot of regulated genes when compare the mesothelial cells of early and late stage. **c,** Heatmap shows the expression level of toll like receptors in early and late stages of the disease. **(d)** represents incoming (receptor) signaling, and **(e)** represents outgoing (ligand) signaling. EC: endothelial cell; Mac/mono: macrophage/monocyte; FB: fibroblast; CM: cardiomyocyte; PC: pericyte; T: T cell; Endo: endocardial cells; Neu: neutrophil SMC: smooth muscle cell: LC: lymphatic cell; B: B cell; Mes: mesothelial cell; NC:  neuron cell; Adipo: adipocyte.

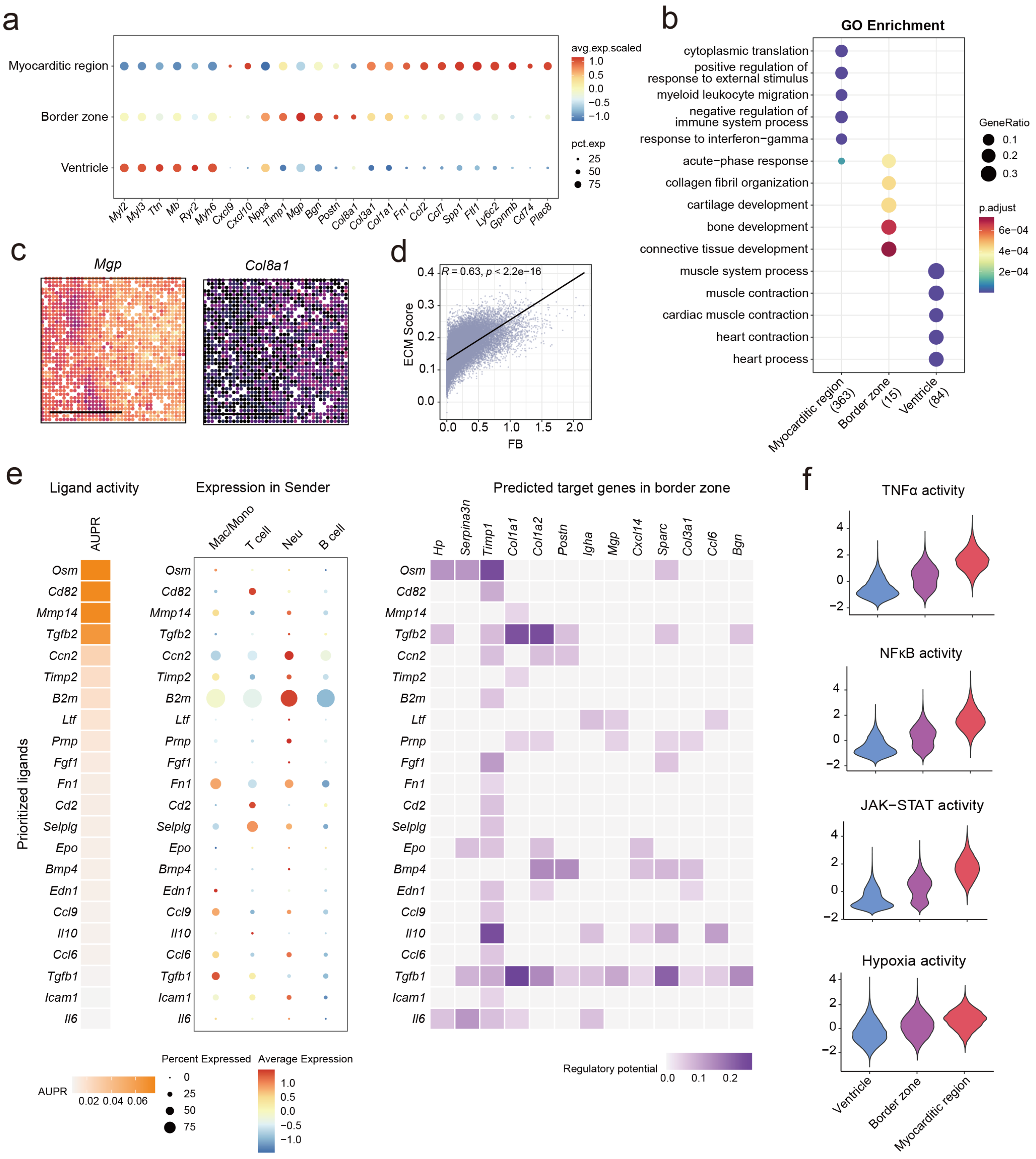


**Figure. S7. The characteristics of border zone. a,** Dot plot showing the expression levels of characteristic genes in each spatial area. **b,** Dot plot showing the representative pathways of three distinct spatial region. **c,** Spatial distribution of *Mgp* (left) and *Col8a1* (right) on spatial section. **d,** Correlation between FB and ECM score. **e,** The regulation of immune cells on fibroblast of border zone at the later stage of the disease. **f,** Changes in characteristic pathways across the infected ventricle.

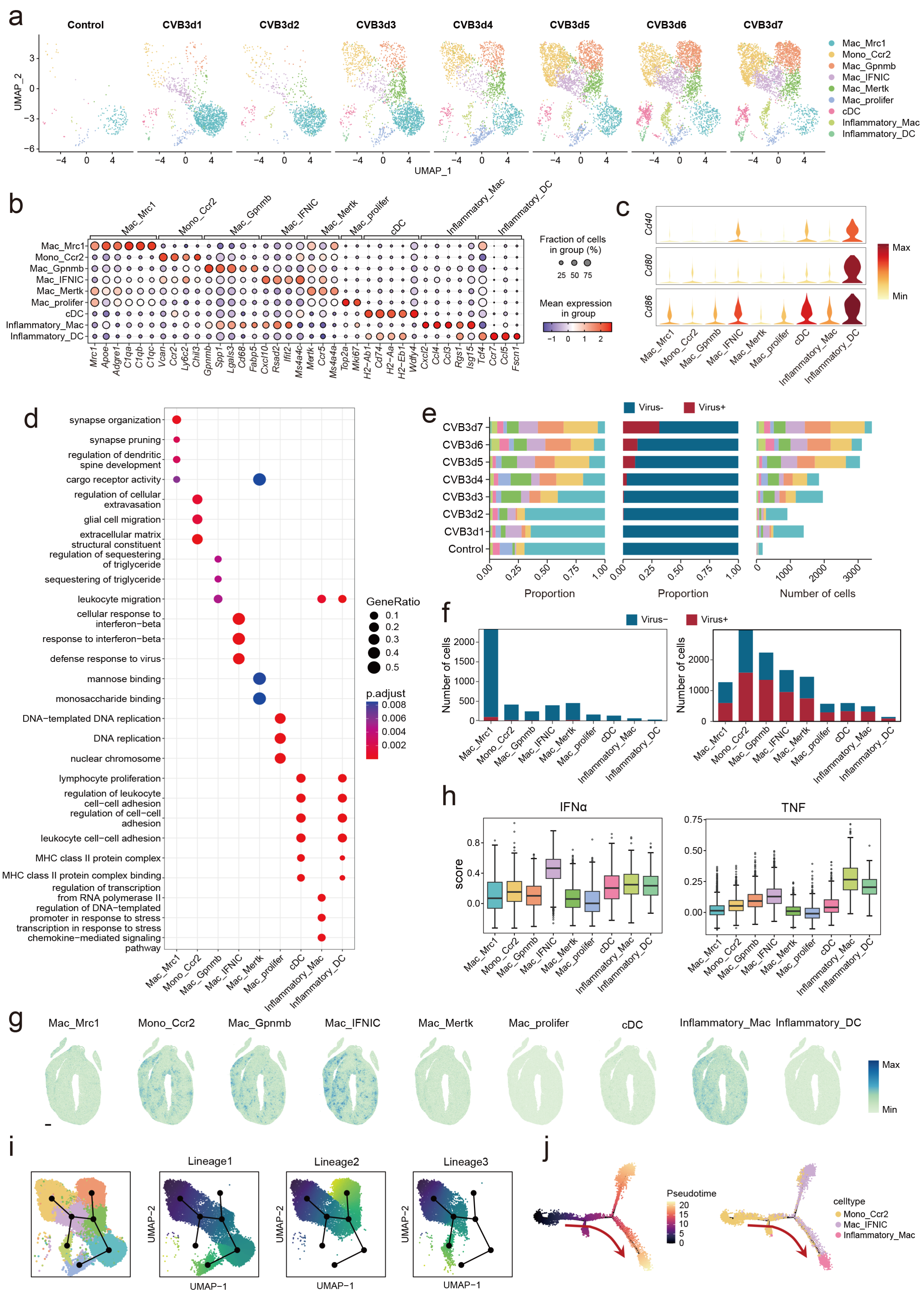
a
Control
CVB3d1
CVB3d2
CVB3d3
CVB3d4
CVB3d5
CVB3d6
CVB3d7
UMAP_1
UMAP_2
Mac_Mrc1
Mono_Ccr2
Mac_Gpnmb
Mac_IFNIC
Mac_Mertk
Mac_prolifer
cDC
Inflammatory_Mac
Inflammatory_DC
b
Fraction of cells in group (%)
Mean expression in group
c
Cd40
Cd80
Cd86
Max
Min
d
GeneRatio
p.adjust
synapse organization
synapse pruning
regulation of dendritic spine development
cargo receptor activity
regulation of cellular extravasation
glial cell migration
extracellular matrix structural constituent
regulation of sequestering of triglyceride
sequestering of triglyceride
leukocyte migration
cellular response to interferon−beta
response to interferon−beta
defense response to virus
mannose binding
monosaccharide binding
DNA−templated DNA replication
DNA replication
nuclear chromosome
lymphocyte proliferation
regulation of leukocyte cell−cell adhesion
regulation of cell−cell adhesion
leukocyte cell−cell adhesion
MHC class II protein complex
MHC class II protein complex binding
regulation of transcription from RNA polymerase II
regulation of DNA−templated promoter in response to stress
transcription in response to stress
chemokine−mediated signaling pathway
e
Virus-
Virus+
Proportion
Number of cells
f
Number of cells
h
IFNα
TNF
score
g
i
Lineage1
Lineage2
Lineage3
UMAP−1
UMAP−2
j
Pseudotime
celltype

**Figure. S8. Cell typing and characteristics of cardiac macrophages. a,** UMAP embedding of all the cells colored by cell clusters from control and CVB3-infected mice heart from 1 to 7 dpi. **b,** Dot plot showing the expression levels of cell-typing genes in each cell cluster. The color represents the gene expression level. **c,** Violin plots showing the showing the expression levels of costimulatory genes in each macrophages cell cluster. **d,** Dot plot showing the representative pathways of each cell cluster. **e,** Proportion changes of macrophage clusters among different time points (left), proportion changes of CVB3 positive macrophages among different time points (middle) and the cell number change of macrophage clusters among different time points (right). **f,** Bar plots showing the number of CVB3 positive macrophages at early stage (left) and late stage (right). **g,** The spatial distribution of macrophage subpopulations on cardiac sections of representative CVB3-infected mice heart at 7 dpi. Scale bar: 500μm. **h,** Box plots showing the score of IFNα and TNF in each cell cluster. **i,** UMAP embedding shows three developmental trajectory of macrophage subclusters calculated by Slingshot. **j,** Pseudotime trajectory colored by pseudotime (left) and cell types (right) initiated from Mono_Ccr2, towards Mac_IFNIC and Inflammatory_Mac predicted by Monocle2.

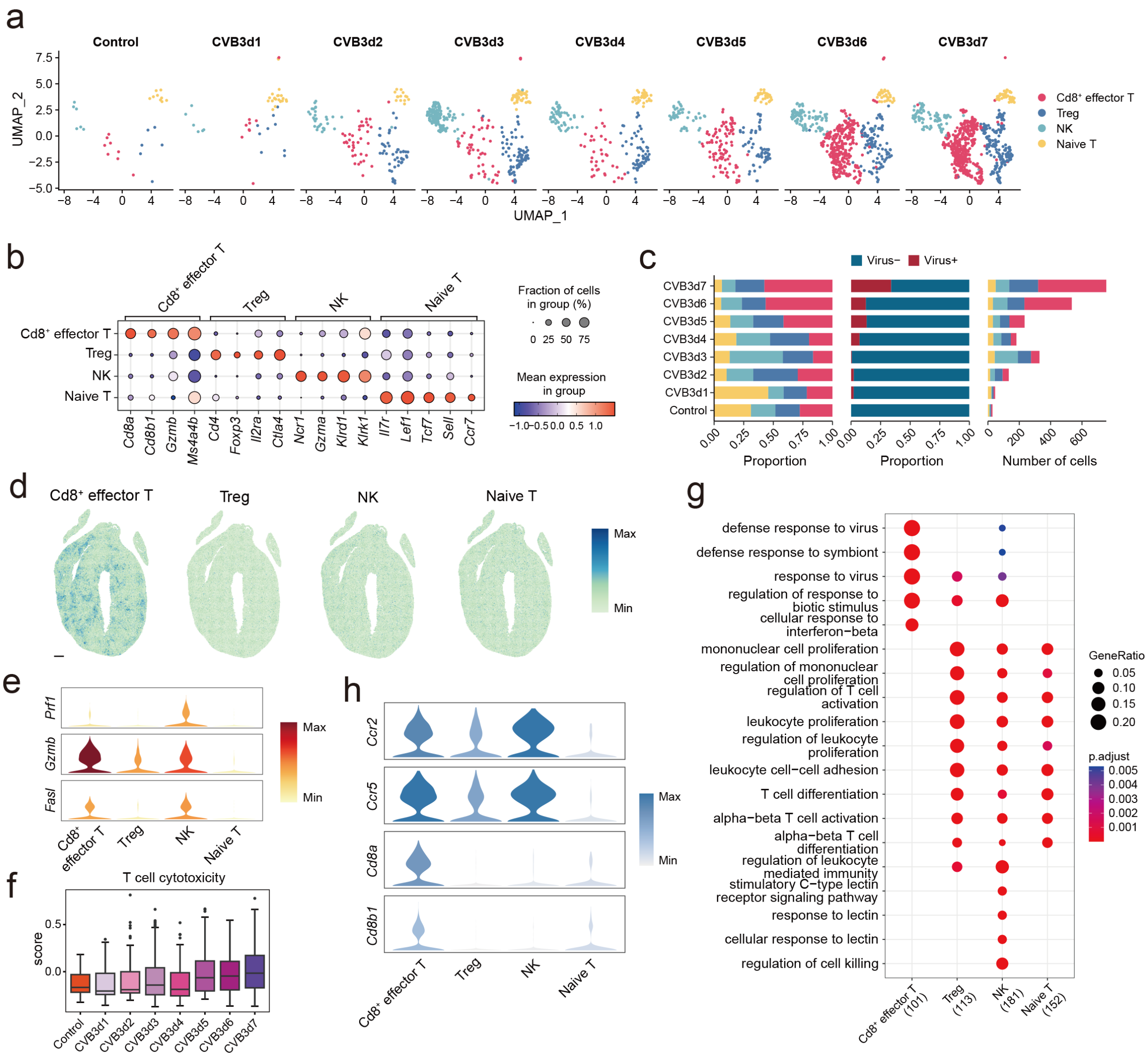


**Figure. S9. Cell typing and characteristics of cardiac T cells. a,** UMAP embedding of all the T cells colored by cell clusters from control and CVB3-infected mice heart from 1 to 7 dpi. **b,** Dot plot showing the expression levels of cell-typing genes in each cell cluster. The color represents the gene expression level. **c,** Proportion changes of T cell clusters among different time points (left), proportion changes of CVB3 positive T cells among different time points (middle) and the cell number change of T cell clusters among different time points (right). **d,** The spatial distribution of T cell subpopulations on cardiac sections of representative CVB3-infected mice heart at 7 dpi. Scale bar: 500μm. **e,** Violin plot showing the expression levels of cytotoxic genes in each T cell cluster. The color represents the gene expression level. **f,** Box plot showing the score of T cell cytotoxicity in T cells at different time points. **g,** Dot plot showing the representative pathways of each cell cluster. **h,** Violin plot showing the expression levels of representative genes in each T cell cluster.

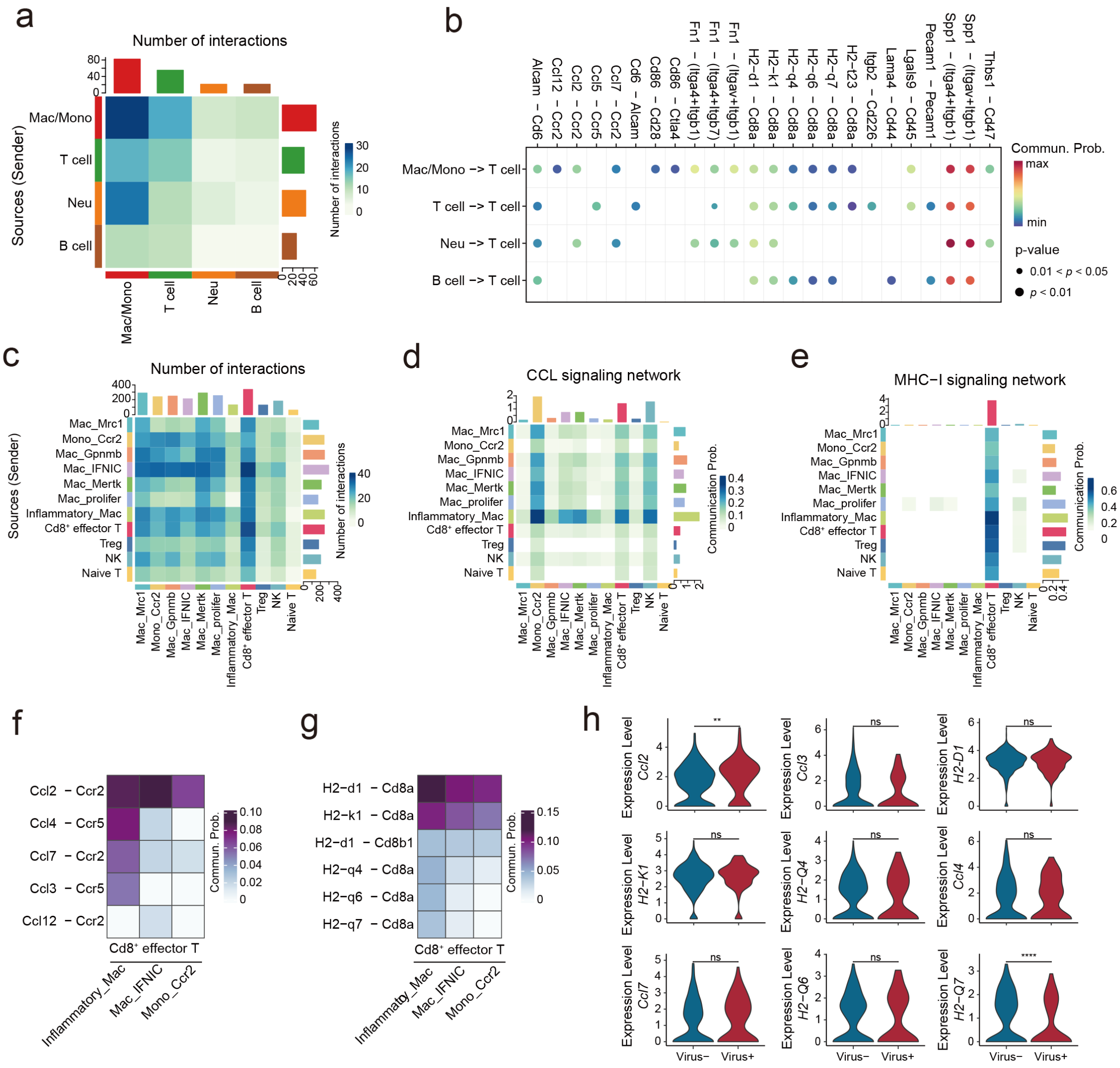


**Figure. S10. Cell interaction among immune cells. a,** Heatmap showing the number of signaling interaction among immune cells. **b,** Bubble plot showing significant ligand-receptor interaction pairs from immune cells to T cells. **c,** Heatmap showing the number of signaling interaction among macrophage subpopulations and T cell subpopulations. **d,** Heatmap showing the chemotaxis effects among cardiac macrophage subpopulations and T cell subpopulations through Ccl–Ccr. **e,** Heatmap showing the interactions among cardiac macrophage subpopulations and T cell subpopulations through MHC-I signaling. Heatmap showing the chemotaxis effects between cardiac macrophage subpopulations of trajectory 3 and CD8 effector T cells through Ccl–Ccr **(f)** and MHC-I molecules **(g)**. **h,** Violin plot shows the expression of Ccl and MHC-I molecules in CVB3 positive and CVB3 negative macrophages. Mac/mono: macrophage/monocyte; T: T cell; Neu: neutrophil B: B cell.

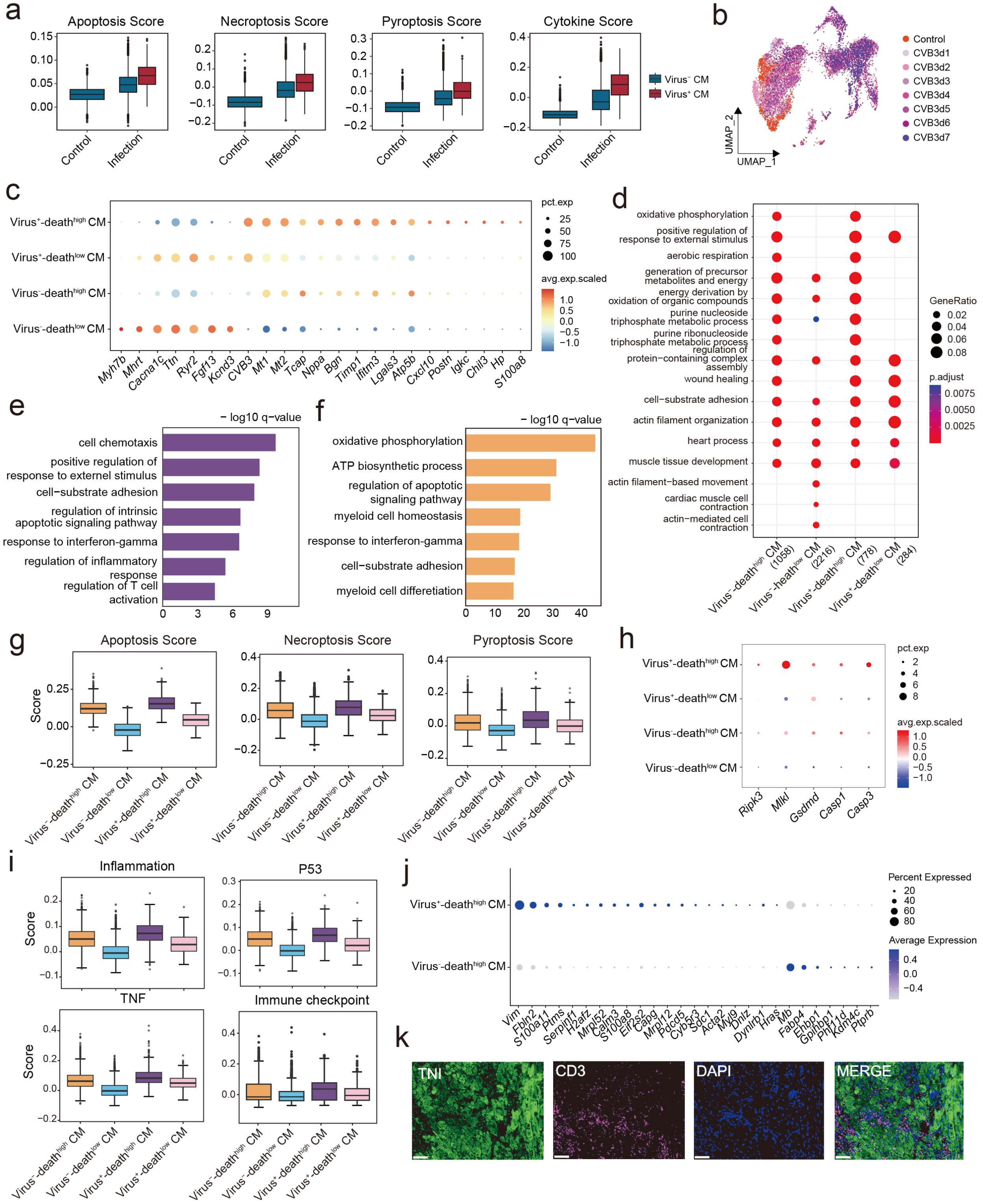


**Figure. S11. Cell typing and characteristics of cardiomyocytes. a,** Box plots of apoptosis score (left), necroptosis score (middle left), pyroptosis score (middle right) and cytokine score (right) among cardiomyocytes of control group, CVB3 negative cardiomyocytes of CVB3 infected group

and CVB3 positive cardiomyocytes of CVB3 infected group. **b,** UMAP embedding of all the cardiomyocytes colored by samples. **c,** Dot plot showing the expression levels of cell-typing genes in each cell cluster. The color represents the gene expression level. **d,** Dot plot showing the representative pathways of each cell cluster. Top pathways of virus$^{-}$-death$^{low}$ CMs (e) and virus$^{-}$-death$^{high}$ CMs **(f)**. **g,** Box plots of apoptosis score (left), necroptosis score (middle) and pyroptosis score (right) among different cardiomyocyte subclusters. Scores were calculated by AddModuleScore function of the Seurat package. **h,** Dot plot of key death genes expression in four types of CMs. **i,** Box plots of inflammation score (left top), P53 (right top), TNF (left bottom) and immune inhibit checkpoint score (right bottom) among different cardiomyocyte subclusters. **j,** Dot plot showing the different death-related genes expression in virus$^{-}$-death$^{high}$ CMs and virus$^{+}$-death$^{high}$ CMs. **k,** Immunofluorescence staining of cardiomyocytes and T cells. TNI: marker of cardiomyocytes; CD3: marker of T cells; DAPI: nucleus. Scale bar: 50μm.

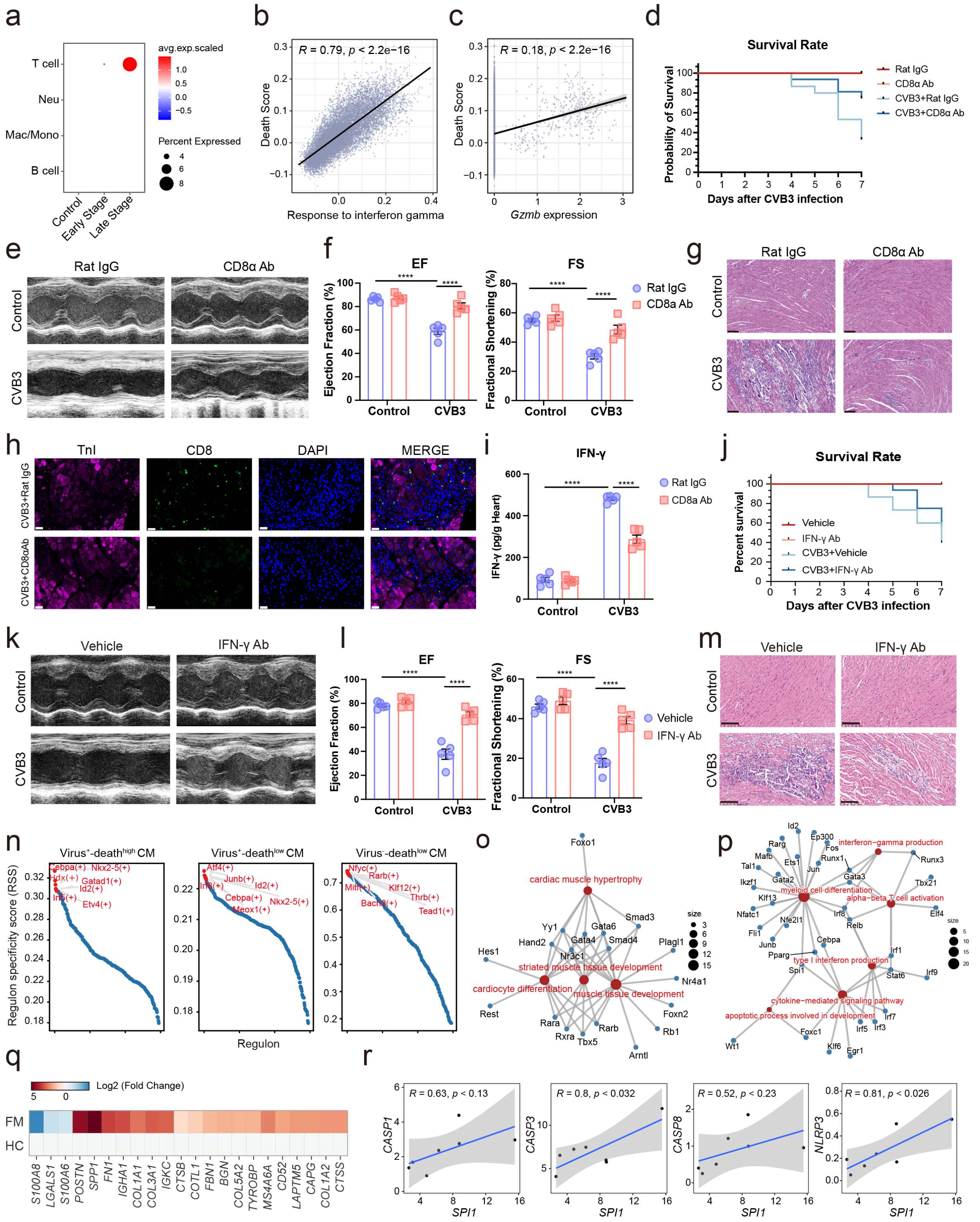


**Figure. S12. Cardiomyocyte death is associated with IFN-γ and blockade of IFN-γ improves cardiac function. a,** The expression level of *Ifng* in immune cells at different stages. **b,** Correlation of response to interferon gamma score with death score of cardiomyocytes. **c,** Correlation of *Gzmb*

expression with death score of cardiomyocytes. Survival rate **(d),** cardiac functions (ejection fraction, fraction shortening, representative echocardiography images **(e),** ejection fraction, and fraction shortening changes (n=5 per group, data are represented as mean ± SEM) **(f)** and H&E staining images **(g)** of CD8α antibody-treated mice. Scale bar: 100μm. **h,** Immunofluorescence staining of cardiomyocytes and $Cd8^+$ T cells of FM and CD8α antibody-treated mice. Scale bar: 20μm. TNI: marker of cardiomyocytes; CD8: marker of $Cd8^+$ T cells; DAPI: nucleus. **i,** the level of IFN-γ in the heart of FM and CD8α antibody-treated mice (n=5 per group, data are represented as mean ± SEM). Survival rate **(j)**, representative echocardiography images **(k),** cardiac function changes (n=5 per group, data are represented as mean ± SEM) **(l)** and H&E staining images **(m)** of FM and IFN-γ antibody-treated mice. Scale bar: 100μm. **n,** The top regulons that activated in three types of cardiomyocytes. The regulon modulatory network of virus$^-$-death$^{low}$ CMs **(o)** and virus$^-$-death$^{high}$ CMs **(p)**. **q,** The expression of high expression genes in myocarditic region in HC (n = 3) and FM patients (n = 4). **r,** Correlation of *SPI1* and death genes (*CASP1*, *CASP3*, *CASP8* and *NLRP3*) in HC (n = 3) and FM patients (n = 4). HC: healthy; FM: fulminant myocarditis.

a

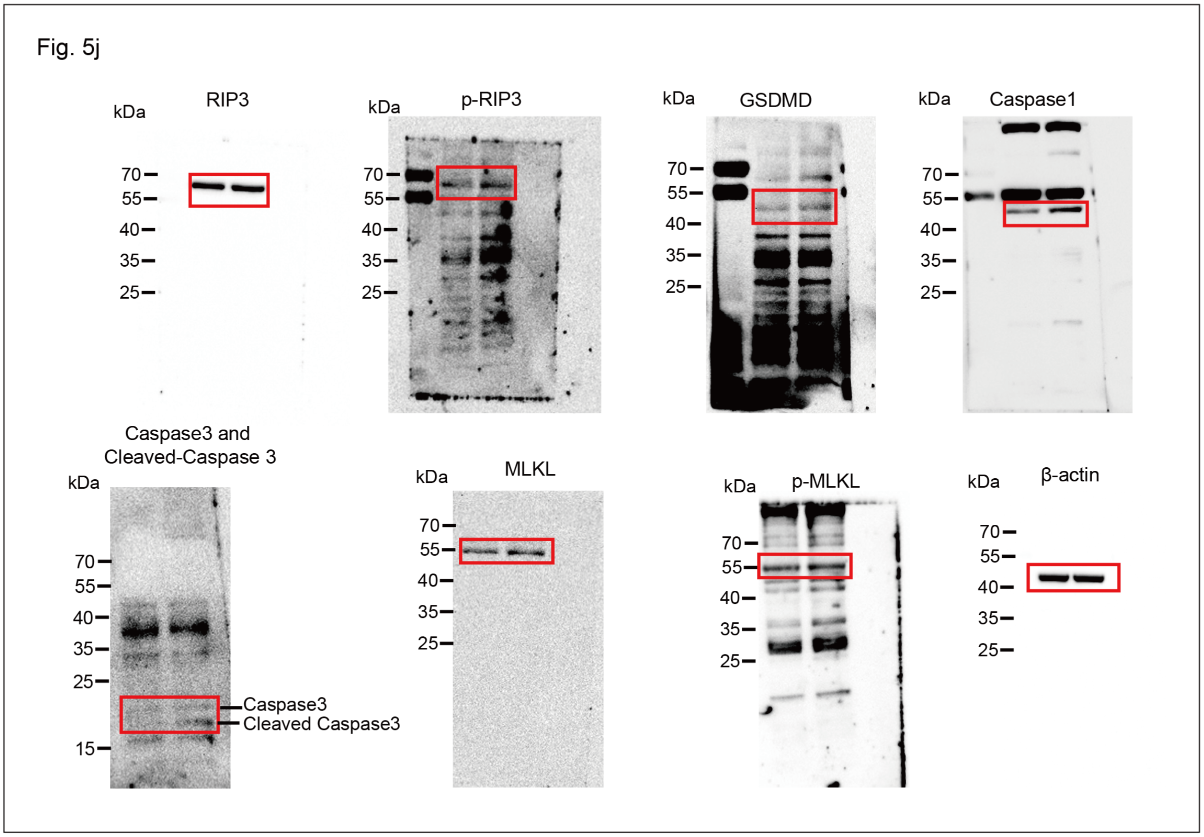
Fig. 5j
RIP3
p-RIP3
GSDMD
Caspase1
Caspase3 and Cleaved-Caspase 3
Caspase3
Cleaved Caspase3
MLKL
p-MLKL
β-actin
kDa
70
55
40
35
25
15

b

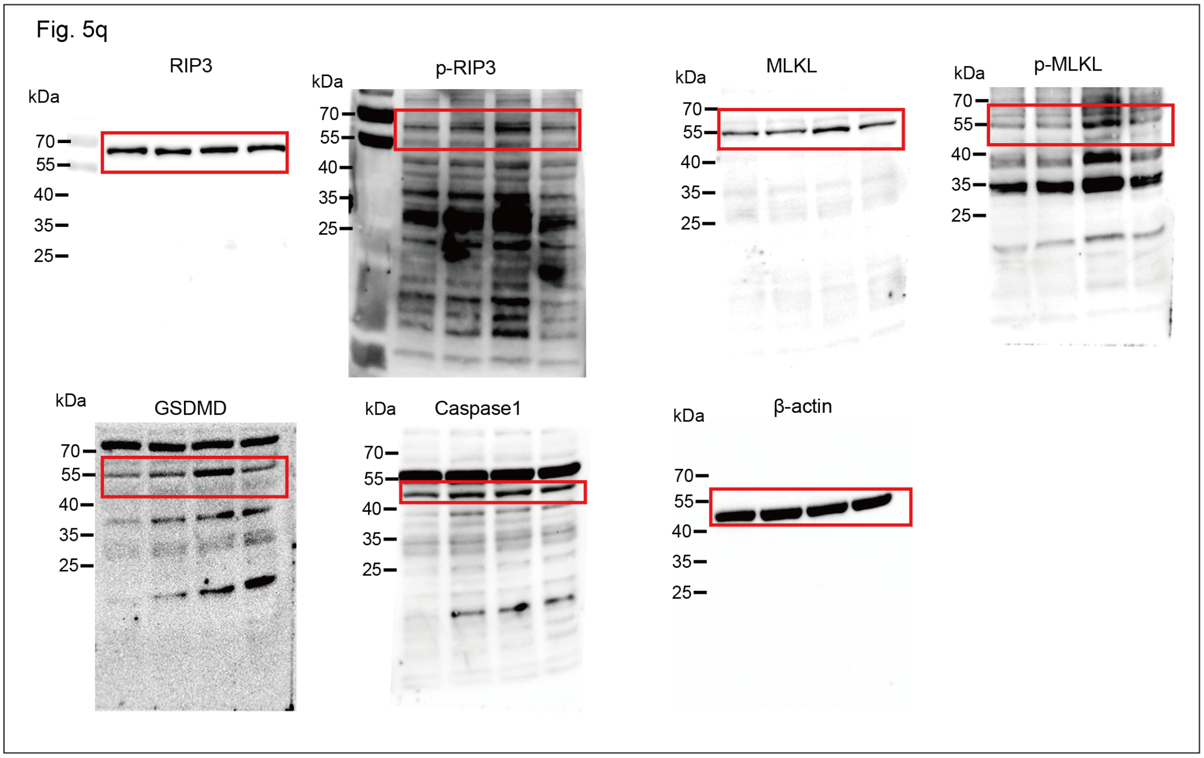
Fig. 5q
RIP3
p-RIP3
MLKL
p-MLKL
GSDMD
Caspase1
β-actin
kDa
70
55
40
35
25

**Figure. S13. Original and uncropped films of Western blots. a,** Original and uncropped western blot mambranes of Fig.5j. **b,** Original and uncropped western blot mambranes of Fig.5q.

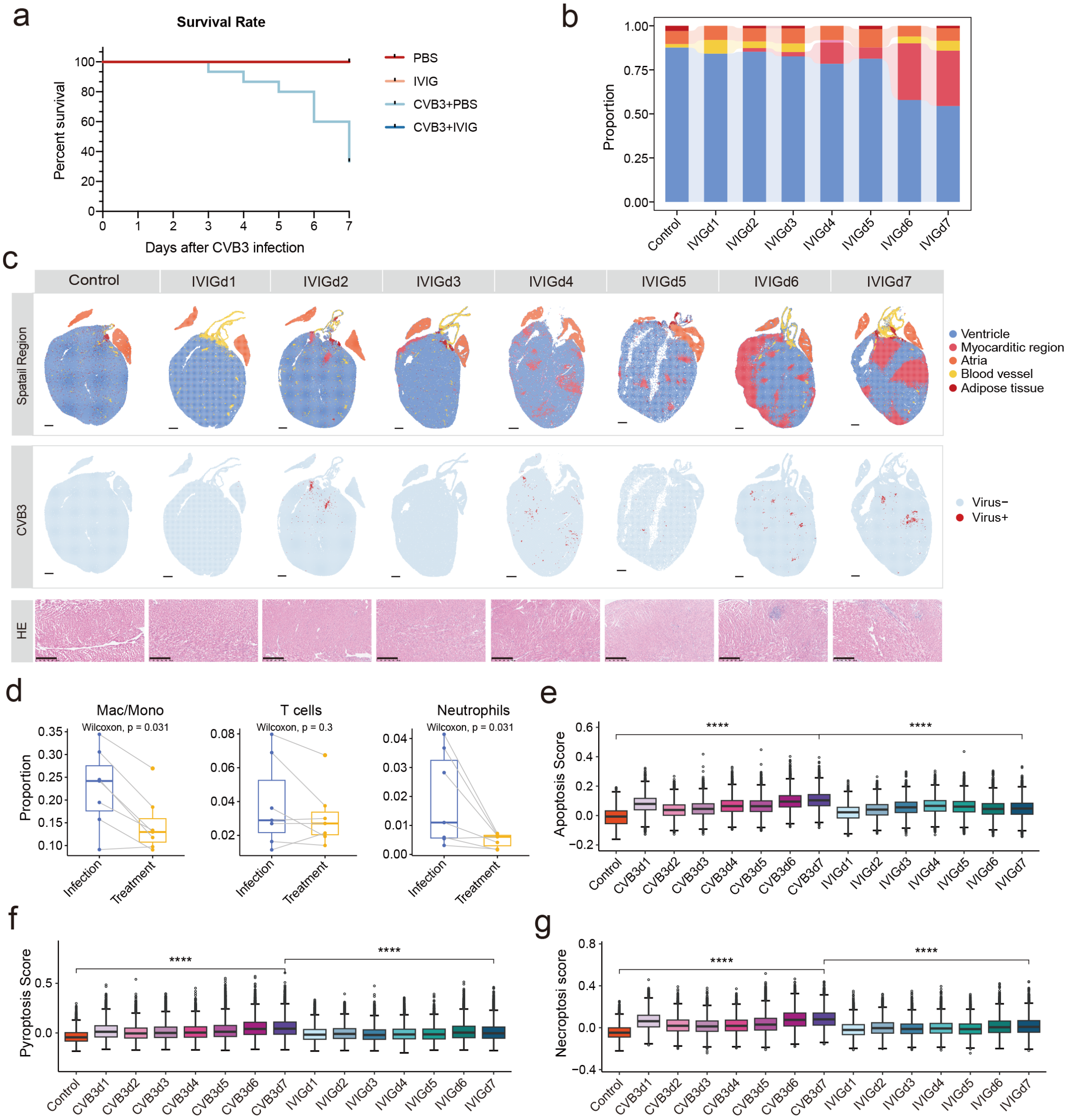

**Figure. S14. Treatment effects of intravenous immunoglobulin (IVIG) on mice with FM. a,** Survival rate of FM and IVIG-treated mice. **b,** Spatial structure changes among different time points. **c,** The spatial transcriptomes (top), CVB3 positive cells (middle) and representative H&E staining (bottom) of cardiac tissue sections from control and IVIG treated FM mice from 1 to 7 dpi. Scale bar: 500µm. **d,** The proportion of Mac/Mono, T cells and neutrophils before and after treatment. The level of apoptosis **(e)**, pyroptosis **(f)** and necroptosis **(g)** score in control and CVB3 infected hearts from 1 to 7 dpi, before and after treatment.
